# Charged Triazole Cross-Linkers for Hyaluronan-Based Hybrid Hydrogels

**DOI:** 10.3390/ma9100810

**Published:** 2016-09-30

**Authors:** Maike Martini, Patricia S. Hegger, Nicole Schädel, Burcu B. Minsky, Manuel Kirchhof, Sebastian Scholl, Alexander Southan, Günter E. M. Tovar, Heike Boehm, Sabine Laschat

**Affiliations:** 1 Institut für Organische Chemie, Universität Stuttgart, Pfaffenwaldring 55, Stuttgart D-70569, Germany; maike.martini@gmx.de (M.M.); nicole.schaedel@oc.uni-stuttgart.de (N.S.); kirchhof.manuel@yahoo.de (M.K.); scholl.kirchheim@freenet.de (S.S.); 2 Department of Cellular Biophysics & CSF Biomaterials, Max-Planck Institute for Medical Research, Heidelberg D-69120, Germany; hegger@is.mpg.de (P.S.H.); minsky@is.mpg.de (B.B.M.); 3 Department of Biophysical Chemistry, University of Heidelberg, Im Neuenheimerfeld 253, Heidelberg D-69120, Germany; 4 Institut für Grenzflächenverfahrenstechnik und Plasmatechnologie IGVP, Universität Stuttgart, Nobelstr. 12, Stuttgart D-70569, Germany; alexander.southan@igvp.uni-stuttgart.de (A.S.); guenter.tovar@igvp.uni-stuttgart.de (G.E.M.T.); 5 Fraunhofer-Institut für Grenzflächen- und Bioverfahrenstechnik IGB, Nobelstr. 12, Stuttgart D-70569, Germany; guenter.tovar@igb.fraunhofer.de

**Keywords:** hyaluronan, triazole, triazolium, hydrogels, cross-linking

## Abstract

Polyelectrolyte hydrogels play an important role in tissue engineering and can be produced from natural polymers, such as the glycosaminoglycan hyaluronan. In order to control charge density and mechanical properties of hyaluronan-based hydrogels, we developed cross-linkers with a neutral or positively charged triazole core with different lengths of spacer arms and two terminal maleimide groups. These cross-linkers react with thiolated hyaluronan in a fast, stoichiometric thio-Michael addition. Introducing a positive charge on the core of the cross-linker enabled us to compare hydrogels with the same interconnectivity, but a different charge density. Positively charged cross-linkers form stiffer hydrogels relatively independent of the size of the cross-linker, whereas neutral cross-linkers only form stable hydrogels at small spacer lengths. These novel cross-linkers provide a platform to tune the hydrogel network charge and thus the mechanical properties of the network. In addition, they might offer a wide range of applications especially in bioprinting for precise design of hydrogels.

## 1. Introduction

Hyaluronan is a naturally occurring linear polysaccharide containing an alternating sequence of glucuronic acid and *N*-acetylglucosamine units. It is a crucial constituent of the extracellular matrix and contributes to the unique properties of connective tissue and cartilage. Therefore, hyaluronan and synthetically modified hyaluronan derivatives are of high interest for polymer chemistry, materials science, and regenerative medicine [1,2,3]. In particular, hydrogels from unmodified hyaluronan or thiolated hyaluronan (HA-SH) have been extensively studied [4,5,6]. The cross-linking of HA-SH with poly(ethylene glycol) diacrylate [7,8] or poly(ethylene glycol) vinylsulfone [9] is a well known strategy for tailoring the properties of hydrogels.

It is known that the charge density on the polymer and the ionic strength in aqueous media influence the hydrogel properties such as the swelling ratio and the elastic modulus [10,11,12]. For hydrogels and nanoparticles, the negative charge of polyanions such as hyaluronan can be utilized by (a) ionic cross-linking with polycations such as polyallylamine hydrochloride or modified chitosan [13,14,15]; (b) ionic cross-linking with a low molecular weight cation [16]; (c) covalent cross-linking with a polycation or a cationic dendrimer [17]; or (d) photochemical cross-linking of methacrylate-functionalized HA with an unsaturated polycation [18]. These materials provide various applications such as gene transfection [17], biosensors for enzymatic reactions [16], and films for uni-directional drug delivery and controlled release [13]. While several cross-linking methodologies have been developed [19], short, low-molecular-weight cross-linkers consisting of a rigid heterocycle and flexible tethers carrying reactive groups have rarely been employed for this purpose. This approach should lead to a toolbox for the creation of sets of hydrogels with a wide range of rheological properties by simply adjusting the length of the tethers and the charge density on the heterocyclic core.

**Figure 1:**
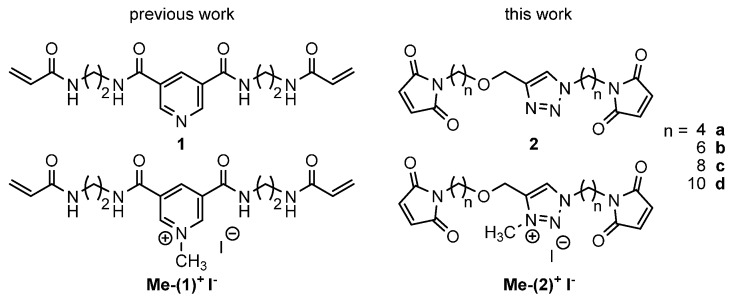
Low-molecular-weight cross-linkers with pyridine or triazole cores.

We recently developed desmosine-inspired cross-linkers **1** and **Me-(1)^+^ I^−^** with a 3,5-diacyl-pyridine or pyridinium core tethered to two terminal acrylamide units (Figure 1). These pyridine derivatives were used as cross-linkers for thiolated hyaluronan via thio-Michael addition to provide hyaluronan-based hydrogels [20,21]. Alternatively, they were also successfully applied to a synthetic piperazinyl-modified poly(ethylene glycol) derivative via aza-Michael addition to give the corresponding hydrogels [22]. Expanding this concept of short, low-molecular-weight cross-linkers consisting of a rigid heterocycle with flexible tethers carrying reactive groups, we developed a novel class of cross-linkers bearing triazole cores (Figure 1). These neutral triazole cross-linkers **2** and their corresponding triazolium salts **Me-(2)^+^ I^−^** allow for higher synthetic flexibility and the independent modification of both tethers.

1,2,3-Triazoles are available via Cu(I)-catalyzed azide-alkyne cycloaddition (CuAAC) or the corresponding copper-free reactions and have been recognized as highly versatile building blocks for a variety of functional materials [23,24]. They have also been used to generate hyaluronan-based hydrogels [25,26,27]. First, hyaluronan was chemically modified to bear azide and alkyne groups and subsequently cross-linked via the click-reaction. Thus, the triazole is formed during the final gelation step.

We wanted to utilize the stability of HA-hydrogels cross-linked via triazoles and take it one step further. Therefore, we designed low-molecular-weight cross-linkers already containing a triazole core as well as two Michael acceptor groups, maleimides. This approach enables us to alkylate the triazole ring prior to cross-linking; a positive charge can thereby be introduced into the hydrogel structure. The final charged or neutral cross-linkers react with thiolated hyaluronan via the thio-Michael addition. Finally, this approach allows us to compare structurally similar neutral triazole-based cross-linkers **2** and positively charged triazolium cross-linkers **Me-(2)^+^ I^−^** with regard to their ability to form hydrogels with thiolated hyaluronan and their influence on the hydrogel properties.

## 2. Results and Discussion

### 2.1. Synthesis of Cross-Linkers **2** and **Me-(2)^+^ I^−^**

**Scheme 1:**
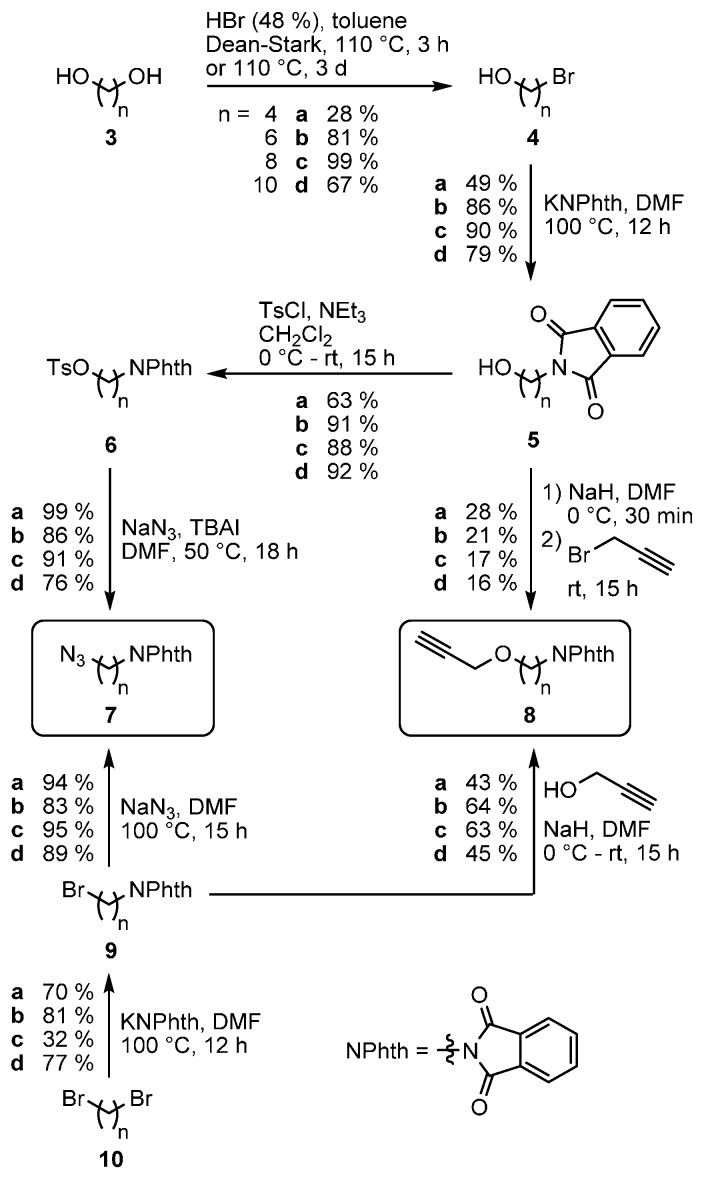
Synthesis of triazole precursors **7** and **8** by different synthetic strategies.

The synthesis of novel triazole cross-linkers **2** and **Me-(2)^+^ I^−^** commenced with the preparation of the azide and alkyne precursors **7** and **8** (Scheme 1 and see Supplementary Materials Scheme S1).

Following the method by Kress [28], 1,ω-diols **3** were treated with HBr in toluene under Dean Stark conditions to provide the bromoalcohols **4** in good yields, except for 4-bromobutanol **4a** due to the formation of THF as a by-product. Subsequent nucleophilic substitution with potassium phthalimide gave the hydroxy phthalimides **5** with a 49%–90% yield [29]. The tosylation of the hydroxy phthalimides **5** and displacement with NaN_3_ following procedures from Yi [30] and Tran [31] provided the *N*-phthalimido-protected azides **7** in 62%–80% over two steps. The synthesis of the alkyne precursors **8** could also be achieved from the *N*-phthalimido alcohols **5** following a procedure from Tran [31] by treatment with NaH and propargylbromide in moderate yields of 16%–28%.

As an alternative strategy towards the azides **7** and alkynes **8**, we investigated a two-step route starting from the dibromides **10**. First, they were converted to the *N*-phthalimido bromides **9** according to Kong [32], followed by either S_N_2 reaction [33] to the azides **7** with an 83%–95% yield or by Williamson etherification [31] with propargylic alcohol to the alkynes **8** with a 43%–64% yield.

Comparison of the overall yields for the triazole precursors **7** and **8** by the two strategies shows that the synthesis from the dibromides **10** is preferable, as it comprises less reaction and purification steps, and the yields are usually higher.

**Scheme 2:**
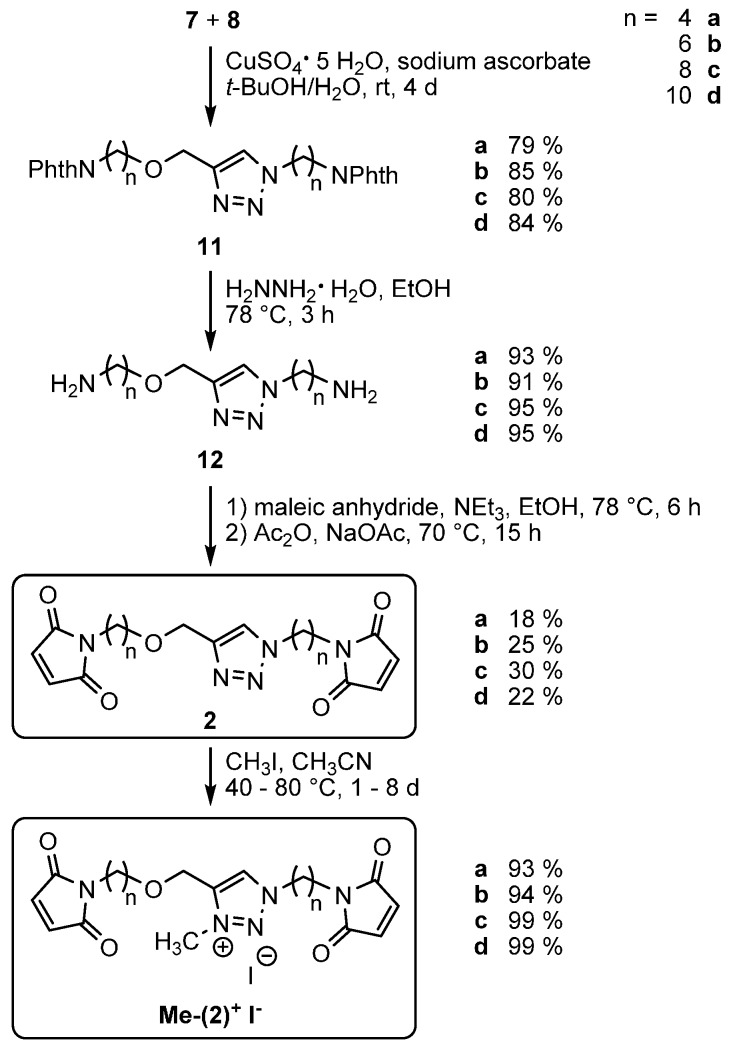
Synthesis of the triazole cross-linkers **2** and **Me-(2)^+^ I^−^**.

The CuAAC of azides **7** and alkynes **8** was performed according to Sharpless conditions [34] with CuSO_4_ and sodium ascorbate in a *tert*-butanol/water mixture to provide the *N*‑phthalimido-terminated triazoles **11** with a 79%–85% yield (Scheme 2). Subsequent hydrazinolysis [35] proceeded uneventfully, and the resulting diaminotriazoles **12** were converted to the bismaleimidotriazoles **2** by treatment with maleic anhydride and NEt_3_, followed by reaction with Ac_2_O and NaOAc according to the procedure by Liu [36] with a 17%–29% yield over three steps. The yield for the maleimide cross-linkers was quite low due to the formation of several by-products during the last two steps, such as the addition of AcOH to the maleimides. This already shows the high reactivity of maleimides in Michael addition reactions. Finally, *N*-methylation with methyl iodide [37] provided the corresponding triazolium salts **Me-(2)^+^ I^−^** in high yields.

**Scheme 3:**
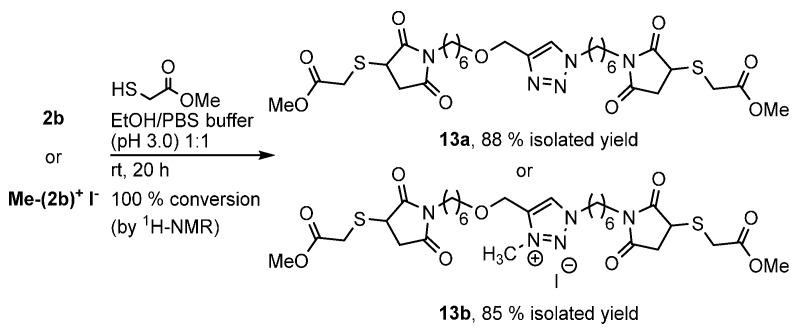
Proof-of-concept thio-Michael addition reaction of **2b** and **Me-(2b)^+^ I^−^** with methyl thiogylcolate.

The ability of the novel cross-linkers **2b** and **Me-(2b)^+^ I^−^** to undergo thio-Michael addition reactions at the maleimide units was shown in vitro with methyl thioglycolate in ethanol and phosphate buffer solution (PBS) (pH 3.0) at room temperature (Scheme 3). In order to obtain a complete conversion, 1.0 equiv of cross-linkers **2b** or **Me-(2b)^+^ I^−^** and 2.5 equiv of methyl thioglycolate were used. After a 20-h reaction time, the Michael addition products **13a** and **13b** could be isolated with high yields of 88% and 85%. Monitoring the reaction by NMR revealed a rapid conversion of the starting material within 10 min (Figures S1 and S2).

### 2.2. Formation of Hydrogels with Thiolated Hyaluronan

In the next step, we tested the ability of our novel cross-linkers to form hydrogels with thiolated hyaluronan. Therefore, we employed well-characterized, research-grade hyaluronan with an average molecular weight of 125 kDa (contour length (*L*) ≈ 301 nm) and synthetically modified the carboxylic groups with a short thiol linker leading to a statistical thiolation degree of 40% (HA_125_-SH_40_).

Generally, the rate of thio-Michael addition reactions can be adjusted by the choice of reactive groups and catalytic additives [38]. To enhance the reaction rate compared to our previously described cross-linkers **1** and **Me-(1)^+^ I^−^** bearing acrylamides, we introduced maleimides as reactive groups in **2** and **Me-(2)^+^ I**^−^. Maleimides are expected to lead to the highest reaction kinetics in thio-Michael additions [38].

**Figure 2:**
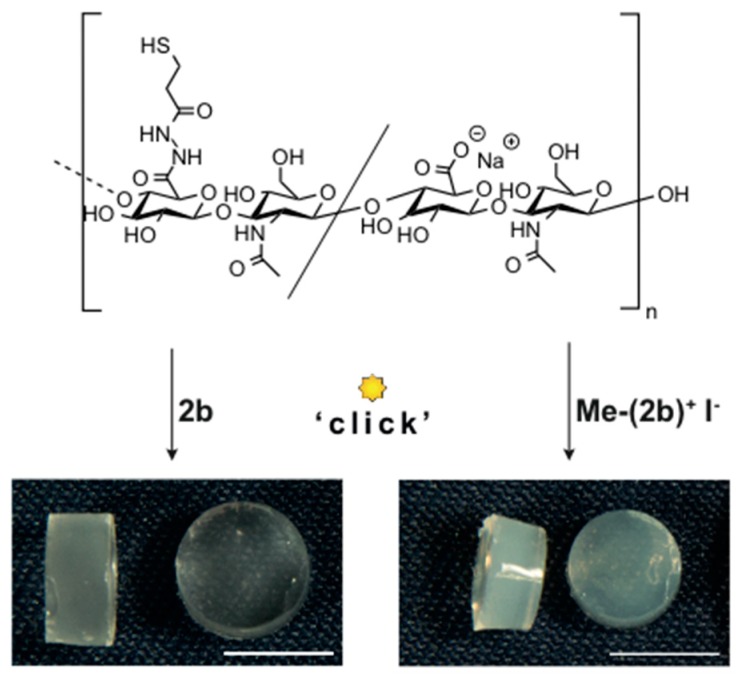
Form-stable, semi-opaque hydrogels can be obtained with, e.g., bismaleimidotriazole cross-linker **2b** and **Me-(2b)^+^ I^−^** and statistically thiolated hyaluronan. Here, we employ hyaluronan with an average weight of 125 kDa (HA_125_-SH_40_), corresponding to an average number of 330 disaccharide monomers. Scale bars represent 0.5 cm.

Therefore, all cross-linking reactions were carried out with relatively short hyaluronan chains, to favor intermolecular cross-links, at a relatively low pH of 3.0. At these conditions, all of the hydrogels were formed in less than five minutes, leading to gelation times ideally suited where fast polymerization is desired. The rheological properties of the obtained hydrogels were further characterized in bulk hydrogels with an 8-mm diameter. Generally, form-stable hydrogels are obtained with **2a–c** and **Me-(2a–d)^+^ I^−^** respectively (exemplary gels with **2b** and **Me-(2b)^+^ I^−^** are shown in Figure 2). However, bismaleimido-triazoles **2** and **Me-(2)^+^ I^−^** and HA_125_-SH_40_ almost instantaneously cross-link into very inhomogeneous gels and were thus not analyzed further. Due to the harsh reaction conditions, stability of HA_125_ and HA_125_-SH_40_ under these conditions was confirmed by agarose gel electrophoresis (Figure S3), and the results showed that the length of both HA_125_ and HA_125_-SH_40_ does not change.

The E-moduli of the hydrogels of HA_125_-SH_40_ cross-linked with **2a–c**, **Me-(2a–d)^+^ I^−^** were both dependent on the spacer lengths *n* and the charge of the cross-linker core (Table 1). For neutral triazole cross-linkers **2a,b** with short C_4_ or C_6_ spacers, similar E-moduli were obtained. Upon increasing the spacer length, the E-modulus decreased by one order of magnitude for triazole **2c** with C_8_ spacer. Triazole **2d** with C_10_ spacer did not even lead to stable gel formation. In contrast, the E-moduli of hyaluronan-hydrogels carrying the charged cross-linkers **Me‑(2a–d)^+^ I^−^** were not influenced significantly by the length of the spacer. In particular triazolium cross-linker **Me‑(2d)^+^ I^−^** with a C_10_ spacer showed similar E-moduli, as compared with the homologues with shorter chain lengths.

Based on the defined thio-Michael reaction, all gels had a very similar number of cross-links, as seen by the similar amount of reacted thiols in each hydrogel (Table 1). Considering the similar degree of cross-linking, additional charge interactions most likely enhance the E-modulus of gels with charged cross-linkers (Figure S4). These additional electrostatic interactions seem to exceed the effect of spacer-length in HA_125_-SH_40_-**Me-(2a–d)^+^ I^−^** gels.

Next, swelling ratios of the hydrogels (wet weight/dry weight) were measured in PBS and water (Table 2). Generally, the swelling ratio is two times larger in water as compared to PBS irrespective of spacer lengths or charge of the cross-linker. This is in agreement with results from various polyelectrolyte gels where the swelling behavior was explained by interactions of the charged moieties in the cross-linked polymer chains and dissolved ions [39,40,41]. For hydrogels with neutral triazole cross-linkers **2a–c**, the swelling ratio reveals no clear trend (Table 2). On the contrary, for the hydrogels carrying triazolium units, the swelling ratio in water decreased with the increasing chain length, i.e., from 68 for **Me-(2a)^+^ I^−^** with C_4_ spacer to 19 for **Me-(2d)^+^ I^−^** with C_10_ spacer. This indicates a strong ionic interaction between hyaluronan hydrogels cross-linked with positively charged cross-linkers, also represented by the calculation of mesh sizes (ξ) from the swelling ratios by

(1) 1Mc = 2Mn− (νV1)[ln(1− ν2,s)+ ν2,s+ χν2,s2]ν2,r×[(ν2,sν2,r)13−12×(ν2,sν2,r)] 

(2) $$\xi \;={\nu_{2,s}}^{-\frac{1}{3}}-\;\sqrt{\frac{2\;C_{n}\;M_{c}}{M_{r}}\;}\;l$$

M_c_ = molecular weight between two adjacent crosslinks;

M_n_ = number-average degree of polymerization = 125,000 (average molecular weight of HA);

ν = specific volume of bulk HA = 0.764 cm^3^/g;

V_1_ = molar volume of solvent (assumed to be the same as water) = 18 cm^3^/mol;

ν_2,s_ = equilibrium swollen polymer volume fraction;

ν_2,r_ = unswollen polymer volume fraction;

χ = Flory-Huggins interaction parameter for HA in water = 0.439;

C_n_ = characteristic ratio of HA = 27;

M_r_ = molecular weight of the HA repeat (disaccharide) unit = 415 g/mol;

l = length of a virtual bond (defined from glycosidic oxygen to glycosidic oxygen, spanning a monosaccharide) = 0.52 nm.

## 3. Materials and Methods

### 3.1. Synthesis of Maleimide Cross-Linkers **2** and **Me-(2)^+^ I^−^**

The synthesis and characterization of all reported compounds can be found in the Supplementary Materials.

### 3.2. Thiolation of HA and Ellman’s Assay

Thiolation of sodium hyaluronate with an average moleculare weight of 125 kDa (HA_125_, Lifecore Biomedical) was carried out with 3,3’-Dithiobis(propanoic dihydrazide) at the carboxyl group [42]. 3,3’-Dithiobis(propanoic dihydrazide) was synthesized according to the procedure described in [43]. Reaction time for thiolation was chosen to yield an intermediate thiolation degree of around 40% of carboxyl groups (HA_125_-SH_40_), analyzed by Ellman’s assay [44].

### 3.3. HA-Hydrogel Formation

All solutions used for hydrogel formation were degassed for 15 min in an ultrasonic bath to avoid disulfide bond formation. The HA_125_-SH_40_ was dissolved in PBS, pH = 7.4, resulting in a 4% (w/v) HA_125_-SH_40_ solution at a final pH of 3.0. All cross-linkers were dissolved in 70% EtOH in different concentrations to obtain a 1:0.8 ratio of thiol vs. maleimide in the resulting hydrogel. A mixture of 70% HA_125_-SH_40_ and 30% cross-linker solutions was prepared, to obtain a final HA_125_-SH_40_ concentration of 2.8% (w/v) in the hydrogel. Immediately after mixing, gelation solutions were poured into small cylindrical Teflon molds (r = 3 mm, h = 3 mm). Molds were sealed with glass slides, and gelation was allowed to proceed for 24 h at room temperature. Gels were swollen in PBS for 48 h at room temperature to reach equilibrium.

### 3.4. Mechanical Testing

Mechanical properties of the swollen gels were measured with the NanoBionix Universal Testing System (MTS Systems Corp., Oak Ridge, TN, USA) in uniaxial compression mode with parallel plate geometry. Thereby, an increasing strain from 0% to 10% was applied to the gels, and the resulting forces were measured. The data was analyzed in the linear-viscoelastic region between 0% and 5% compression by a linear fit of the stress-strain curves. All values represent the mean value of three independent experiments, and the errors show standard deviation.

Swelling ratios were determined by dividing the swollen weight by the dry weight of the hydrogels: Swollen weights were measured after swelling the hydrogels in PBS and double-distilled water (ddH_2_O) for 48 h at room temperature. Subsequently, hydrogels were freeze-dried for three days in a lyophilizer (JUMO IMAGO 500, Pietkowski-Forschungsgeräte, Munich, Germany) to determine the dry weight. Mesh sizes were subsequently estimated using the method established by Peppas and Merrill [45]. All measurements were done in triplicate and are shown as mean with standard deviation as errors.

## 4. Conclusions

In conclusion, we have herein demonstrated that neutral and charged short, low-molecular weight cross-linkers **2** and **Me-(2)^+^ I^−^** can be obtained in a convergent fashion via CuAAC from the azides **7** and alkynes **8**. This approach provides rapid access to cross-linkers of various chain lengths. It should be noted that the CuAAC is particularly useful for asymmetrical cross-linkers, which are not so easily accessible via the double functionalization of symmetrical heterocyclic precursors. Furthermore, synthesis of triazole cross-linkers independent of the polymerization reaction enables us to introduce an additional charge. Utilizing the stoichiometric thio-Michael addition, a direct comparison of the neutral compounds **2a–c** and the charged **Me-(2a–d)^+^ I^−^** in the cross-linking of thiolated hyaluronan was possible. This provided soft, viscoelastic hydrogels with E-moduli up to 25 kPa. In particular for cross-linkers with longer spacers (C_8_ and C_10_), the charge state of the central heterocyclic unit had a stabilizing impact on the E-moduli. With triazole **2d**, no stable gels were formed; however, upon introduction of a positive charge in **Me-(2d)^+^ I^−^**, hydrogels with an E-modulus of 17 ± 9 kPa were obtained. These hydrogels may be suitable for 3D or bioprinting applications for the precise controlling of topography to “recapitulate” the structural properties of the target tissue.

## Figures and Tables

**Table 1:** Mechanical measurements of HA_125_-SH_40_-**2a–c** and HA_125_-SH_40_-**Me-(2a–d)^+^ I^−^** hydrogels. The ratio of reacted thiols was determined by an adapted Ellman’s assay. All values represent mean and standard deviation of three different experiments. ^1^

Hydrogel	Spacer Length *n*	E-Modulus (kPa)	Reacted Thiols (%)
HA_125_-SH_40_-2a	4	14.60 ± 3.60	85 ± 3
HA_125_-SH_40_-2b	6	14.53 ± 7.07	84 ± 2
HA_125_-SH_40_-2c	8	1.16 ± 0.85	83 ± 4
HA_125_-SH_40_-Me-(2a)^+^ I^−^	4	25.32 ± 10.06	87 ± 4
HA_125_-SH_40_-Me-(2b)^+^ I^−^	6	12.15 ± 3.41	85 ± 3
HA_125_-SH_40_-Me-(2c)^+^ I^−^	8	17.41 ± 8.60	84 ± 3
HA_125_-SH_40_-Me-(2d)^+^ I^−^	10	16.99 ± 8.99	85 ± 4

^1^ The cross-linking of HA_125_-SH_40_ and neutral triazole **2d** with spacer length *n* = 10 did not provide stable gels.

**Table 2:** Swelling ratios (wet weight/dry weight) and mesh sizes (in phosphate buffer solution (PBS)) of HA_125_-SH_40_-**2a–c** and HA_125_-SH_40_-**Me-(2a–d)^+^ I^−^** hydrogels. All values represent mean and standard deviation of three different experiments. ddH_2_O: double-distilled water.

Hydrogel	Spacer Length *n*	Swelling Ratio (PBS)	Swelling Ratio (ddH_2_O)	Mesh Size (PBS) (nm)
HA_125_-SH_40_-2a	4	79.63 ± 1.16	162.37 ± 45.16	51.82 ± 26.31
HA_125_-SH_40_-2b	6	30.22 ± 17.83	68.89 ± 44.36	61.52 ± 10.02
HA_125_-SH_40_-2c	8	100.58 ± 49.91	208.85 ± 134.57	55.81 ± 27.77
HA_125_-SH_40_-Me-(2a)^+^ I^−^	4	67.70 ± 5.79	139.79 ± 28.15	83.93 ± 9.64
HA_125_-SH_40_-Me-(2b)^+^ I^−^	6	49.47 ± 8.63	98.05 ± 9.90	157.84 ± 44.00
HA_125_-SH_40_-Me-(2c)^+^ I^−^	8	33.75 ± 5.75	59.30 ± 13.14	55.94 ± 8.59
HA_125_-SH_40_-Me-(2d)^+^ I^−^	10	18.79 ± 4.14	31.22 ± 9.13	27.16 ± 6.77

## Supplementary Materials

The following are available online at www.mdpi.com/1996-1944/9/10/810/s1: synthesis and characterization of all reported compounds; additional experiments regarding hydrogel characterization.

## Abbreviations

The following abbreviations are used in this manuscript:

HA	hyaluronan, hyaluronic acid
HA-SH	thiolated hyaluronan
HA_125_-SH_40_	hyaluronan with an average molecular weight of 125 kDa and a thiolation degree of 40%
CuAAC	Cu(I)-catalyzed azide-alkyne cycloaddition
NPhth	*N*-phthalimido
rt	room temperature
Me	methyl
Et	ethyl
Ac	acetyl
equiv	equivalents
PBS	phosphate buffered saline
DMF	*N*,*N*-dimethylformamide
Ts	tosyl (protecting group)
TBAI	tetrabutylammonium iodide
ddH_2_O	double-distilled water
