# Supplementary material for: Charged Triazole Cross-Linkers for Hyaluronan-Based Hybrid Hydrogels
Source: Materials (Basel). 2016 Sep 30;9(10):810. doi: 10.3390/ma9100810 (PMC5456633; doi:10.3390/ma9100810)

Supplementary Materials: Charged Triazole Cross-Linkers for Hyaluronan-Based Hybrid Hydrogels

Maike Martini, Patricia S. Hegger, Nicole Schädel, Burcu B. Minsky, Manuel Kirchhof, Sebastian Scholl, Alexander Southan, Günter E. M. Tovar, Heike Boehm and Sabine Laschat

1. Synthesis of Cross-Linkers (2) and Me-(2)$^{+}$ I^{-}

1.1. Synthetic Strategies

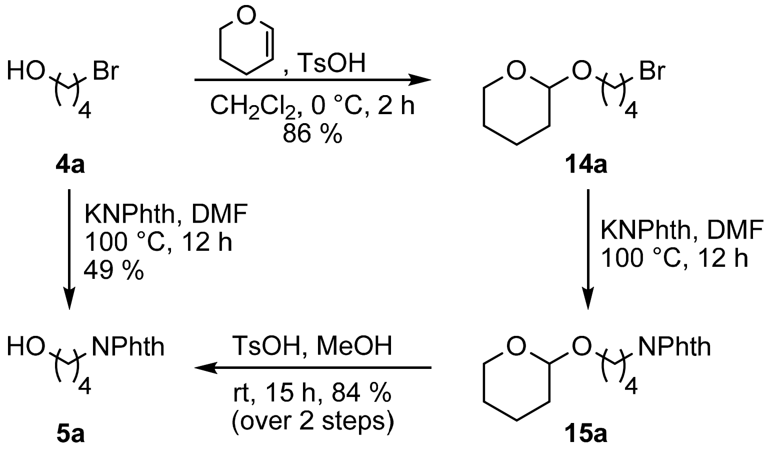

Scheme S1. Additional synthesis route for C_4 precursors.

1.2. General Methods

Melting points were measured with a Stuart SMP10 apparatus and are uncorrected. NMR spectra were recorded on a Bruker Avance 500 at 500 MHz (^{1}H) and at 125 MHz (^{13}C) and on a Bruker Avance 300 at 300 MHz (^{1}H) and at 75 MHz (^{13}C) with tetramethylsilane as an internal standard. The signals were assigned by using additionally HSQC-, COSY-, and HMBC experiments. IR spectra were recorded using ATR technique on a Bruker FT-IR spectrometer Vektor 22 with MKII Golden Gate Single Reflection Diamant ATR system. Mass spectra and HR-MS spectra were recorded on a micro-TOF-Q (Bruker Daltonics), a Finnigan MAT 95, and a Varian MAT 711 spectrometer Varian MAT 711 spectrometer Varian MAT 711 spectrometer. Column chromatography was performed using silica gel 60 (Fluka, grain size 40–63 μm). TLC was performed on Merck Kieselgel 60 F254 plates (0.25 mm thickness on aluminium), and visualized with anisaldehyde reagent (2.00 mL anisaldehyde dissolved in 200 mL conc. HAc and 4.00 mL conc. H_2SO_4) or permanganate reagent (3.00 g $KMnO_4$, 20.0 g K_2CO_3, and 5.00 mL of a 5%-ic NaOH solution in 300 mL H_2O). All chemicals were used as purchased unless otherwise stated. CH_2Cl_2 and NEt_3 were dried over CaH_2 by heating at reflux and subsequent distillation. DMF was stored over molecular sieves 4 Å. Hexanes (b.p. 30–75 °C), EtOAc, CH_2Cl_2 and MeOH used for chromatography were distilled prior to use. Moisture sensitive reactions were performed in oven-dried glassware under N_2 atmosphere. For easier comparison of NMR spectra atom numbering deviates in some cases from the IUPAC nomenclature.

1.3. General Procedures

Synthesis of Bromoalcohols (4) (GP 1)

To a solution of the appropriate diol **3** (1.00 mmol) in toluene (2 mL), HBr (48% in H_2O, 1.20 mmol) was added, and the reaction mixture was heated at reflux for 3 d or for 3 h using a Dean-Stark apparatus. Diethyl ether (1 mL) was added and the mixture was then successively washed with aqueous NaOH (6 M, 1 mL), HCl (3 M, 1 mL), and brine (1 mL). The organic layer was dried (Na_2SO_4) and concentrated. The residue was purified by column chromatography on SiO_2.

Synthesis of Phthalimides (5) (GP 2a)

A solution of the appropriate bromoalcohol **4** (1.00 mmol) and potassium phthalimide (1.20 mmol) in DMF (1 mL) was heated at reflux for 16 h. The precipitate was filtered off and CH_2Cl_2 (3 mL) and H_2O (3 mL) were added. The layers were separated and the aqueous layer was extracted with CH_2Cl_2 (2 × 3 mL) and EtOAc (2 × 3 mL). The combined organic layers were washed with H_2O (5 mL), dried (Na_2SO_4) and concentrated. The residue was purified by column chromatography on SiO_2.

Synthesis of Phthalimides (9) (GP 2b)

A stirred solution of potassium phthalimide (1.00 mmol) and the appropriate α,ω-dibromoalkane **10** (5.00 mmol) in abs. DMF (1 mL) was heated at 100 °C for 16 h. The precipitate was filtered off, and the filtrate was concentrated. For n = 4, 6, the residue was distilled under vacuum (p ≈ 20 mbar) to remove excess dibromoalkane prior to purification by column chromatography on SiO_2. For n = 8, 10 the crude product was purified by column chromatography on SiO_2.

Synthesis of Alkynes (8) (GP 3)

To a suspension of NaH (1.20 mmol) in abs. DMF (2 mL) in an oven-dried Schlenk flask at 0 °C, the appropriate alcohol **5** (1.00 mmol) in abs. DMF (2 mL) was added, followed by the dropwise addition of a solution of propargyl bromide (80% in toluene, 1.20 mmol). Alternatively, propargyl alcohol (1.20 mmol) was added to a suspension of NaH (1.15 mmol) in abs. DMF (1 mL) at 0 °C; after 1 h, a solution of the appropriate bromide **9** (1.00 mmol) in DMF (2.5 mL) was slowly added dropwise. The reaction mixture was stirred at room temperature for 16 h. Then, the solvent was removed under vacuum and the residue taken up in CH_2Cl_2 (5 mL). The solid was filtered off and the filtrate concentrated. The residue was purified by column chromatography on SiO_2.

Synthesis of Tosylates (6) (GP 4)

To a solution of the appropriate alcohol **5** (1.00 mmol) in abs. CH_2Cl_2 (2.5 mL) in an oven-dried Schlenk flask at 0 °C, abs. NEt_3 (2.20 mmol) and *p*-toluenesulfonyl chloride (1.30 mmol) were added, and the reaction mixture was stirred at room temperature for 16 h. Then, the mixture was washed with aqueous HCl (1 M, 2.5 mL), and brine (2.5 mL). The aqueous layer was extracted with CH_2Cl_2 (3 × 5 mL), and the combined organic layers were dried (Na_2SO_4) and concentrated. The residue was purified by column chromatography on SiO_2.

Synthesis of Azides (7) (GP 5a)

A solution of the appropriate tosylate **6** (1.00 mmol), TBAI (0.10 mmol), and NaN_3 (1.10 mmol) in abs. DMF (10 mL) was heated at 50 °C for 16 h. After removal of the solvent under vacuum, the residue was taken up in Et_2O (10 mL), the solid filtered off, the filtrate concentrated and purified by column chromatography on SiO_2.

Synthesis of Azides (7) (GP 5b)

A solution of the appropriate bromide **9** (1.00 mmol) and NaN_3 (1.50 mmol) in DMF (5.5 mL) was stirred at 100 °C for 16 h. For n = 4, the reaction mixture was poured onto ice water. The colorless precipitate was filtered off and dried under vacuum. For n > 4, the solvent was evaporated, and the residue was taken up in Et_2O (30 mL) and filtered. The filtrate was concentrated and purified by column chromatography on SiO_2.

Synthesis of Triazoles (11) (GP 6)

To a solution of the appropriate azide **7** (1.00 mmol) and alkyne **8** (1.00 mmol) in a mixture of *t*-BuOH/H_2O (1:1, 10 mL), $CuSO_4{\cdot}5\ H_2O$ (0.01 mmol) and sodium ascorbate (0.10 mmol) were added, and the reaction mixture was stirred at room temperature for 4 d. After the addition of CH_2Cl_2 (7 mL), the layers were separated and the aqueous layer was extracted with CH_2Cl_2 (3 × 5 mL). The combined extracts were dried (Na_2SO_4) and concentrated. The residue was purified by column chromatography on SiO_2.

Synthesis of Diamines (12) (GP 7)

A solution of the appropriate diphthalimide **11** (1.00 mmol) und hydrazine hydrate (10.0 mmol) in EtOH (50 mL) was heated at reflux for 3 h. Precipitated 2,3-dihydrophthalazine-1,4-dione was filtered off and the filtrate concentrated. The residue was taken up in EtOH (as little as possible) and filtered. This procedure was repeated until a yellow oil remained after concentration.

Synthesis of Maleimides (2) (GP 8)

To a solution of the appropriate diamine **12** (1.00 mmol) in EtOH (40 mL), NEt_3 and maleic anhydride (2.40 mmol each) were successively added, and the reaction mixture was heated at reflux for 6 h. The solvent was removed under vacuum, the residue taken up in Ac_2O (100 mmol) and NaOAc (2.40 mmol) was added. The reaction mixture was heated at 70 °C for 16 h. After the addition of H_2O and CH_2Cl_2 (15 mL each), the layers were separated, and the aqueous layer was extracted with CH_2Cl_2 (3 × 15 mL). The combined organic layers were dried (Na_2SO_4) and concentrated. The residue was purified by column chromatography on SiO_2.

Synthesis of Triazolium Salts (**Me-(2)$^+$ I$^-$**) (GP 9)

A solution of the appropriate triazole **2** (1.00 mmol) und MeI (20.0 mmol) in MeCN (10 mL) was heated at 40 °C for 8 d (for n = 4) and at reflux for 1–4 d (for n > 4). Solvent and excess MeI were distilled off, and the products **Me-(2)$^+$ I$^-$** were isolated in pure form without further purification.

Thio-Michael Reaction of Crosslinkers (2) or (**Me-(2)$^+$ I$^-$)** with a Thiol (GP 10)

To a solution of crosslinker C_6-triazole **2b** or triazolium **Me-(2b)$^+$ I$^-$** (55 μmol) in degassed EtOH (1.5 mL) a degassed PBS solution (pH 3.0, 1.5 mL) was added. Then, methyl thioglycolate (0.14 mmol) was added dropwise, and the reaction mixture was stirred at room temperature for 24 h. The mixture was then extracted with CH_2Cl_2 (3 × 5 mL), the organic layer dried ($MgSO_4$) and the solvent removed under reduced pressure.

1.4. Synthesis of Triazole Precursors (7) and (8)

4-Bromobutan-1-ol (4a)

According to GP 1, from butane-1,4-diol (10.2 g, 0.11 mol), HBr (48% in H_2O, 15.4 mL, 23.0 g, 0.14 mol), toluene (100 mL), chromatography with hexanes/EtOAc (5:1, then 3:1), yield: 4.77 g, 31.2 mmol, 28%, yellow oil; R_f = 0.32 (hexanes/EtOAc 3:1). ^{1}H-NMR (300 MHz, $CDCl_3$): δ = 1.70–1.81 (m, 2H, CH_2), 1.89–2.01 (m, 2H, CH_2), 3.44 (t, *J* = 6.5 Hz, 2H, 4-H), 3.75 (t, *J* = 6.4 Hz, 2H, 1-H) ppm.

^{13}C-NMR (75 MHz, $CDCl_3$): δ = 29.1, 30.4 (C-2, C-3), 33.5 (C-4), 62.4 (C-1) ppm. The spectroscopic data are in accordance with those in the literature [1].

6-Bromohexan-1-ol (4b)

According to GP 1, from hexane-1,6-diol (5.08 g, 43.0 mmol), HBr (48% in H_2O, 5.80 mL, 8.70 g, 51.6 mmol), toluene (50 mL), chromatography with hexanes/EtOAc (3:1, then 1:1), yield: 6.29 g, 34.7 mmol, 81%, colorless oil; R_f = 0.24 (hexanes/EtOAc 3:1). ^{1}H-NMR (300 MHz, $CDCl_3$): δ = 1.32–1.50 (m, 4H, 3-H, 4-H), 1.52–1.63 (m, 2H, 2-H), 1.69 (s, 1H, OH), 1.81–1.92 (m, 2H, 5-H), 3.40 (t, *J* = 6.7 Hz, 2H, 6-H), 3.63 (t, *J* = 6.7 Hz, 2H, 1-H) ppm. ^{13}C-NMR (75 MHz, $CDCl_3$): δ = 25.1 (C-3), 28.1 (C-4), 32.6 (C-2), 32.8 (C-5), 34.0 (C-6), 62.9 (C-1) ppm. The spectroscopic data are in accordance with those in the literature [2,3].

8-Bromooctan-1-ol (4c)

According to GP 1, from octane-1,8-diol (10.0 g, 68.4 mmol), HBr (48% in H_2O, 9.35 mL, 13.8 g, 82.1 mmol), toluene (140 mL), yield: 14.2 g, 68.2 mmol, 99%, yellow oil. ^{1}H-NMR (500 MHz, $CDCl_3$): δ = 1.30–1.37 (m, 6H, CH_2), 1.38 (br s, 1H, OH), 1.40–1.47 (m, 2H, CH_2), 1.57 (tt, *J* = 7.0, 6.8 Hz, 2H, 2-H), 1.86 (tt, *J* = 7.1, 7.0 Hz, 2H, 7-H), 3.41 (t, *J* = 7.0 Hz, 2H, 8-H), 3.64 (t, *J* = 6.8 Hz, 2H, 1-H) ppm. ^{13}C-NMR (125 MHz, $CDCl_3$): δ = 25.6 28.1, 28.7, 29.2 (CH_2), 32.7 (C-2), 32.8 (C-7), 34.0 (C-8), 63.0 (C-1) ppm. The spectroscopic data are in accordance with those in the literature [3].

10-Bromodecan-1-ol (4d)

According to GP 1, from decane-1,10-diol (10.0 g, 57.4 mmol), HBr (48% in H_2O, 7.8 mL, 11.6 g, 68.9 mmol), toluene (100 mL), chromatography with hexanes/EtOAc (4:1, then 2:1); yield: 9.12 g, 38.5 mmol, 67%, yellow oil. ^{1}H-NMR (500 MHz, $CDCl_3$): δ = 1.26–1.38 (m, 10H, CH_2), 1.34 (s, 1H, OH), 1.39–1.45 (m, 2H, CH_2), 1.57 (tt, *J* = 7.0, 6.8 Hz, 2H, 2-H), 1.85 (tt, *J* = 7.3, 7.0 Hz, 2H, 9-H), 3.41 (t, *J* = 7.0 Hz, 2H, 10-H), 3.64 (t, *J* = 6.8 Hz, 2H, 1-H) ppm. ^{13}C-NMR (125 MHz, $CDCl_3$): δ = 25.7, 28.2, 28.8, 29.3, 29.4, 29.5 (CH_2), 32.7 (C-2), 32.8 (C-9), 34.0 (C-10), 63.1 (C-1) ppm. The spectroscopic data are in accordance with those in the literature [2,3].

2-(4-Bromobutoxy)tetrahydro-2H-pyran (14a)

To a solution of 4-bromobutan-1-ol **4a** (5.96 g, 38.9 mmol) in abs. CH_2Cl_2 (60 mL) at 0 °C, 3,4-dihydro-2*H*-pyran (4.2 mL, 3.93 g, 46.7 mmol) and *p*-TsOH·H_2O (20.0 mg, 0.11 mmol) were added, and the reaction mixture was stirred at 0 °C for 2 h. After the addition of a satd. $NaHCO_3$ solution (5 mL), the mixture was successively washed with H_2O (50 mL) and brine (50 mL). The aqueous layers were extracted with CH_2Cl_2 (100 mL) and the combined organic extracts dried ($MgSO_4$). The solvent was removed under reduced pressure and the crude product purified by column chromatography on SiO_2 with hexanes/EtOAc (50:1, then 20:1) to give **14a** (7.92 g, 33.4 mmol, 86%) as a colorless oil. R_f = 0.34 (hexanes/EtOAc 20:1). ^{1}H-NMR (500 MHz, $CDCl_3$): *δ* = 1.48–1.62 (m, 4H, 3-H_a, 4-H_a, 5-H), 1.67–1.77 (m, 3H, 3-H_b, 2′-H), 1.78–1.85 (m, 1H, 4-H_b), 1.93–2.01 (m, 2H, 3′-H), 3.42 (dt, *J* = 9.7, 6.2 Hz, 1H, 1′-H_a), 3.45 (t, *J* = 6.7 Hz, 2H, 4′-H), 3.47–3.53 (m, 1H, 6-H_a), 3.76 (dt, *J* = 9.7, 6.4 Hz, 1H, 1′-H_b), 3.81 - 3.88 (m, 1H, 6-H_b), 4.57 (t, *J* = 3.4 Hz, 1H, 2-H) ppm. ^{13}C-NMR (125 MHz, $CDCl_3$): *δ* = 19.8 (C-4), 25.6 (C-5), 28.6 (C-2′), 30.0 (C-3′), 30.9 (C-3), 33.9 (C-4′), 62.5 (C-6), 66.6 (C-1′), 99.0 (C-2) ppm. The spectroscopic data are in accordance with those in the literature [1].

2-[4-(Tetrahydro-2H-pyran-2-yloxy)butyl]-1H-isoindole-1,3(2H)-dione (15a)

According to GP 2a, from **14a** (2.58 g, 10.9 mmol), potassium phthalimide (2.42 g, 13.1 mmol), DMF (20 mL), 120 °C for 15 h. Generally, the product was used without purification, but it can be purified by chromatography with hexanes/EtOAc (5:1); yield: 2.27 g, 7.47 mmol, 69%, colorless oil. R_f = 0.57 (hexanes/EtOAc 3:1); ^{1}H-NMR (500 MHz, $CDCl_3$): *δ* = 1.44–1.60 (m, 4H, 4′-H, 5′-H_a, 6′-H_a), 1.60–1.72 (m, 3H, 3-H, 6′-H_b), 1.72–1.85 (m, 3H, 2-H, 5′-H_b), 3.40 (dt, *J* = 9.7, 6.3 Hz, 1H, 4-H_a), 3.43–3.52 (m, 1H, 3′-H_a), 3.68–3.78 (m, 1H, 4-H_b), 3.72 (t, *J* = 7.2 Hz, 2H, 1-H), 3.78–3.88 (m, 1H,

3′-H_b), 4.56 (t, *J* = 3.4 Hz, 1H, 1′-H), 7.70 (dd, *J* = 5.4, 3.1 Hz, 2H, CH_{Phth}), 7.83 (dd, *J* = 5.4, 3.1 Hz, 2H, CH_{Phth}) ppm. ^{13}C-NMR (125 MHz, $CDCl_3$): *δ* = 19.6 (C-5′), 25.6, 25.7 (C-2, C-4′), 27.2 (C-3), 30.8 (C-6′), 38.0 (C-1), 62.3 (C-3′), 67.0 (C-4), 98.9 (C-1′), 123.3 (2 × CH_{Phth}), 132.3 (2 × C_{Phth}), 134.0 (2 × CH_{Phth}), 168.5 (2 × C=O) ppm. FT-IR (ATR): $\tilde{\nu}$ = 3469 (w), 2940 (w), 2868 (w), 1771 (w), 1704 (s), 1395 (m), 1360 (m), 1119 (m), 1032 (m), 868 (w), 717 (s), 529 (m) cm^{-1}. MS (ESI): *m*/*z* = 326.14 $[M + Na]^+$. HRMS (ESI): calcd. for $C_{17}H_{21}NO_4Na$ 326.1363, found 326.1388 $[M + Na]^+$.

2-(4-Hydroxybutyl)-1H-isoindole-1,3(2H)-dione (5a)

(a) According to GP 2a, from **4a** (4.50 g, 29.4 mmol), potassium phthalimide (6.54 g, 35.3 mmol), DMF (40 mL), 110 °C for 17 h, chromatography with hexanes/EtOAc (2:1, then 1:1); yield: 3.16 g, 14.4 mmol, 49%, colorless oil, >90% by ^{1}H-NMR.

(b) THP-protected bromoalcohol **14a** (4.53 g, 19.1 mmol) and potassium phthalimide (3.73 g, 20.1 mmol) were stirred at 120 °C for 18 h. The precipitate was filtered off, and EtOAc (50 mL) and H_2O (100 mL) were added to the filtrate. The aqueous layer was extracted with EtOAc (3 × 50 mL). The combined organic layers were washed with H_2O (3 × 100 mL), dried ($MgSO_4$), and concentrated. The crude product was dissolved in MeOH (160 mL), and *p*-TsOH·H_2O (145 mg, 0.76 mmol) was added. The reaction mixture was stirred at room temperature for 20 h. Additional *p*-TsOH·H_2O (145 mg, 0.76 mmol) was added, and the reaction mixture was stirred for another 3 d. The solvent was removed, and the residue was purified by column chromatography on SiO_2 with hexanes/EtOAc (2:1 to 1:1) to give **5a** (3.54 g, 16.1 mmol, 84% over 2 steps) as a colorless solid. R_f = 0.19 (hexanes/EtOAc 2:1). ^{1}H-NMR (300 MHz, $CDCl_3$): *δ* = 1.54–1.65 (m, 2H, CH_2), 1.69–1.81 (m, 2H, CH_2), 1.95 (s, 1H, OH), 3.67 (t, *J* = 6.1 Hz, 2H, 4-H), 3.71 (t, *J* = 6.8 Hz, 2H, 1-H), 7.68 (dd, *J* = 5.4, 3.1 Hz, 2H, CH_{Phth}), 7.81 (dd, *J* = 5.4, 3.1 Hz, 2H, CH_{Phth}) ppm. ^{13}C-NMR (75 MHz, $CDCl_3$): *δ* = 25.2, 29.9 (C-2, C-3), 37.8 (C-1), 62.3 (C-4), 123.3 (2 × CH_{Phth}), 132.2 (2 × C_{Phth}), 134.0 (2 × CH_{Phth}), 168.6 (2 × C=O) ppm. The spectroscopic data are in accordance with those in the literature [4].

2-(6-Hydroxyhexyl)-1H-isoindole-1,3(2H)-dione (5b)

According to GP 2a, from **4b** (9.32 g, 51.5 mmol), potassium phthalimide (11.4 g, 61.8 mmol), DMF (52 mL), chromatography with hexanes/EtOAc (1:1), yield: 10.9 g, 44.3 mmol, 86%, colorless solid; R_f = 0.34 (hexanes/ EtOAc 1:1). ^{1}H-NMR (500 MHz, $CDCl_3$): *δ* = 1.33–1.46 (m, 4H, CH_2), 1.53 (s, 1H, OH), 1.54–1.61 (m, 2H, 5-H), 1.69 (tt, *J* = 7.3, 7.3 Hz, 2H, 2-H), 3.64 (t, *J* = 6.5 Hz, 2H, 6-H), 3.69 (t, *J* = 7.3 Hz, 2H, 1-H), 7.71 (dd, *J* = 5.4, 3.1 Hz, 2H, CH_{Phth}), 7.84 (dd, *J* = 5.4, 3.1 Hz, 2H, CH_{Phth}) ppm. ^{13}C-NMR (125 MHz, $CDCl_3$): *δ* = 25.2 (C-3), 26.5 (C-4), 28.6 (C-2), 32.6 (C-5), 37.9 (C-1), 62.9 (C-6), 123.2 (2 × CH_{Phth}), 132.2 (2 × C_{Phth}), 134.0 (2 × CH_{Phth}), 168.6 (2 × C=O) ppm. The spectroscopic data are in accordance with those in the literature [5].

2-(8-Hydroxyoctyl)-1H-isoindole-1,3(2H)-dione (5c)

According to GP 2a, from **4c** (14.2 g, 67.9 mmol), potassium phthalimide (15.1 g, 81.6 mmol), DMF (68 mL), chromatography with hexanes/EtOAc (1:1), yield: 16.9 g, 61.2 mmol, 90%, colorless solid; R_f = 0.48 (hexanes/EtOAc 1:1). M.p. 65 °C. ^{1}H-NMR (500 MHz, $CDCl_3$): *δ* = 1.28–1.38 (m, 8H, CH_2), 1.43 (s, 1H, OH), 1.55 (tt, *J* = 6.8, 6.7 Hz, 2H, 7-H), 1.67 (tt, *J* = 7.1, 7.0 Hz, 2H, 2-H), 3.63 (t, *J* = 6.7 Hz, 2H, 8-H), 3.68 (t, *J* = 7.1 Hz, 2H, 1-H), 7.71 (dd, *J* = 5.3, 3.0 Hz, 2H, CH_{Phth}), 7.84 (dd, *J* = 5.5, 3.0 Hz, 2H, CH_{Phth}) ppm. ^{13}C-NMR (125 MHz, $CDCl_3$): *δ* = 25.6, 26.7, 28.5, 29.1 (CH_2), 29.2 (C-2), 32.7 (C-7), 38.0 (C-1), 63.0 (C-8), 123.2 (2 × CH_{Phth}), 132.2 (2 × C_{Phth}), 133.9 (2 × CH_{Phth}), 168.5 (2 × C=O) ppm. FT-IR (ATR): $\tilde{\nu}$ = 3461 (w), 2929 (m), 2856 (m), 1772 (w), 1705 (s), 1614 (w), 1467 (w), 1437 (w), 1396 (m), 1368 (m), 1187 (w), 1063 (m), 946 (w), 888 (w), 795 (w), 720 (m), 623 (w), 530 (w) cm^{-1}. MS (ESI) *m*/*z* = 298 $[M + Na]^+$, 276 $[M + H]^+$, 258, 160. HRMS (ESI): calcd. for $[C_{16}H_{21}NO_3Na]^+$ 298.1414, found: 298.1418 $[M + Na]^+$. The ^{1}H-NMR spectrum is in accordance with that in the literature [6].

2-(10-Hydroxydecyl)-1H-isoindole-1,3(2H)-dione (5d)

According to GP 2a, from **4d** (9.12 g, 38.5 mmol), potassium phthalimide (8.56 g, 45.2 mmol), DMF (40 mL), chromatography with hexanes/EtOAc (2:1), yield: 9.23 g, 30.4 mmol, 79%, colorless solid, R_f = 0.63 (hexanes/EtOAc 2:1). ^{1}H-NMR (500 MHz, $CDCl_3$): δ = 1.18–1.42 (m, 12H, CH_2), 1.56 (tt, J = 7.0, 6.7 Hz, 2H, 9-H), 1.62 (s, 1H, OH), 1.67 (tt, J = 7.2, 7.0 Hz, 2H, 2-H), 3.63 (t, J = 6.7 Hz, 2H, 10-H), 3.67 (t, J = 7.2 Hz, 2H, 1-H), 7.71 (dd, J = 5.4, 3.1 Hz, 2H, CH_{Phth}), 7.84 (dd, J = 5.4, 3.1 Hz, 2H, CH_{Phth}) ppm. ^{13}C-NMR (125 MHz, $CDCl_3$): δ = 25.7, 26.8 (CH_2), 28.6 (C-2), 29.1, 29.4, 29.5 (CH_2), 32.8 (C-9), 38.1 (C-1), 63.0 (C-10), 123.2 (2 × CH_{Phth}), 132.2 (2 × C_{Phth}), 133.9 (2 × CH_{Phth}), 168.5 (2 × C=O) ppm. The spectroscopic data are in accordance with those in the literature [7].

2-(4-Bromobutyl)-1H-isoindole-1,3(2H)-dione (9a)

According to GP 2b, from 1,4-dibromobutane (24.1 g, 13.3 mL, 111 mmol), potassium phthalimide (4.13 g, 22.3 mmol), DMF (25 mL), chromatography with hexanes/EtOAc (10:1 to 5:1), yield: 4.37 g, 15.5 mmol, 70%, colorless solid; R_f = 0.39 (hexanes/EtOAc 5:1). ^{1}H-NMR (300 MHz, $CDCl_3$): δ = 1.77–1.96 (m, 4H, 2-H, 3-H), 3.43 (t, J = 6.4 Hz, 2H, 4-H), 3.71 (t, J = 6.6 Hz, 2H, 1-H), 7.70 (dd, J = 5.4, 3.2 Hz, 2H, CH_{Phth}), 7.83 (dd, J = 5.4, 3.2 Hz, 2H, CH_{Phth}) ppm. ^{13}C-NMR (75 MHz, $CDCl_3$): δ = 27.3, 29.9 (C-2, C-3), 32.9 (C-4), 37.0 (C-1), 123.4 (2 × CH_{Phth}), 132.1 (2 × C_{Phth}), 134.1 (2 × CH_{Phth}), 168.5 (2 × C=O) ppm. The spectroscopic data are in accordance with those in the literature [8].

2-(6-Bromohexyl)-1H-isoindole-1,3(2H)-dione (9b)

According to GP 2b, from 1,6-dibromohexane (12.7 g, 8.00 mL, 52.0 mmol), potassium phthalimide (1.93 g, 10.4 mmol), DMF (10 mL), chromatography with hexanes/EtOAc (40:1 to 3:1), yield: 2.61 g, 8.41 mmol, 81%, colorless solid; R_f = 0.46 (hexanes/EtOAc 5:1). ^{1}H-NMR (300 MHz, $CDCl_3$): δ = 1.32–1.53 (m, 4H, 3-H, 4-H), 1.62–1.78 (m, 2H, 2-H), 1.84–1.90 (m, 2H, 5-H), 3.38 (t, J = 6.8 Hz, 2H, 6-H), 3.67 (t, J = 7.2 Hz, 2H, 1-H), 7.70 (dd, J = 5.4, 3.2 Hz, 2H, CH_{Phth}), 7.83 (dd, J = 5.4, 3.2 Hz, 2H, CH_{Phth}) ppm. ^{13}C-NMR (75 MHz, $CDCl_3$): δ = 26.1, 27.8, 28.5, 32.7 (C-2, C-3, C-4, C-5), 33.8 (C-6), 38.0 (C-1), 123.3 (2 × CH_{Phth}), 132.2 (2 × C_{Phth}), 134.0 (2 × CH_{Phth}), 168.5 (2 × C=O) ppm. The spectroscopic data are in accordance with those in the literature [8,9].

2-(8-Bromooctyl)-1H-isoindole-1,3(2H)-dione (9c)

According to GP 2b, from 1,8-dibromooctane (21.1 g, 14.3 mL, 77.6 mmol), potassium phthalimide (4.77 g, 25.8 mmol), DMF (120 mL), recrystallization from pentane, chromatography with hexanes/EtOAc (5:1 to 3:1), yield: 2.77 g, 8.19 mmol, 32%, colorless solid; R_f = 0.67 (hexanes/EtOAc 3:1). ^{1}H-NMR (500 MHz, $CDCl_3$): δ = 1.23–1.47 (m, 8H, CH_2), 1.60–1.73 (m, 2H, 2-H), 1.77–1.89 (m, 2H, 7-H), 3.38 (t, J = 6.9 Hz, 2H, 8-H), 3.67 (t, J = 7.3 Hz, 2H, 1-H), 7.70 (dd, J = 5.4, 3.2 Hz, 2H, CH_{Phth}), 7.84 (dd, J = 5.4, 3.2 Hz, 2H, CH_{Phth}) ppm. ^{13}C-NMR (125 MHz, $CDCl_3$): δ = 26.9, 28.2, 28.66, 28.71, 29.1, 32.9 (CH_2), 34.1 (C-8), 38.1 (C-1), 123.3 (2 × CH_{Phth}), 132.3 (2 × C_{Phth}), 134.0 (2 × CH_{Phth}), 168.6 (2 × C=O) ppm. The spectroscopic data are in accordance with those in the literature [9].

2-(10-Bromodecyl)-1H-isoindole-1,3(2H)-dione (9d)

According to GP 2b, from 1,10-dibromo-decane (50.7 g, 169.0 mmol), potassium phthalimide (5.72 g, 30.9 mmol), DMF (360 mL), chromatography with hexanes/EtOAc (6:1), yield: 8.68 g, 23.7 mmol, 77%, colorless solid (>90% by ^{1}H-NMR); R_f = 0.68 (hexanes/EtOAc 6:1). ^{1}H-NMR (300 MHz, $CDCl_3$): δ = 1.18–1.47 (m, 12H, CH_2), 1.54–1.73 (m, 2H, 2-H), 1.77–1.92 (m, 2H, 9-H), 3.40 (t, J = 6.8 Hz, 2H, 10-H), 3.67 (t, J = 7.3 Hz, 2H, 1-H), 7.70 (dd, J = 5.4, 3.2 Hz, 2H, CH_{Phth}), 7.84 (dd, J = 5.4, 3.2 Hz, 2H, CH_{Phth}) ppm. ^{13}C-NMR (75 MHz, $CDCl_3$): δ = 27.0, 28.3, 28.7, 28.8, 29.2, 29.45, 29.47, 33.0 (CH_2), 34.2 (C-10), 38.2 (C-1), 123.3 (2 × CH_{Phth}), 132.3 (2 × C_{Phth}), 134.0 (2 × CH_{Phth}), 168.6 (2 × C=O) ppm. The spectroscopic data are in accordance with those in the literature [8,9].

4-(1,3-Dioxo-1,3-dihydro-2H-isoindol-2-yl)butyl 4-methylbenzenesulfonate (6a)

According to GP 4, from alcohol **5a** (1.04 g, 4.73 mmol), NEt_3 (1.44 mL, 1.05 g, 10.4 mmol), *p*-TsCl (1.17 g, 6.15 mmol), CH_2Cl_2 (10 mL), chromatography with hexanes/EtOAc (5:1 to 3:1), yield: 1.11 g, 2.96 mmol, 63%, colorless solid, R_f = 0.24 (hexanes/EtOAc 3:1). M.p. 112 °C. ^{1}H-NMR (500 MHz, $CDCl_3$): δ = 1.65–1.74 (m, 4H, 2-H, 3-H), 2.42 (s, 3H, CH_3), 3.64 (t, J = 6.3 Hz, 2H, 1-H), 4.05 (t, J = 5.7 Hz, 2H, 4-H), 7.32 (d, J = 8.1 Hz, 2H, CH_{Ts}), 7.71 (dd, J = 5.4, 3.2 Hz, 2H, CH_{Phth}), 7.77 (d, J = 8.1 Hz, 2H, CH_{Ts}), 7.82 (dd, J = 5.4, 3.2 Hz, 2H, CH_{Phth}) ppm. ^{13}C-NMR (75 MHz, $CDCl_3$): δ = 21.7 (CH_3), 24.8, 26.3 (C-2, C-3), 37.2 (C-1), 69.8 (C-4), 123.4 (2 × CH_{Phth}), 128.0 (2 × CH_{Ts}), 130.0 (2 × CH_{Ts}), 132.3 (2 × C_{Phth}), 133.1 (C_{Ts}), 134.1 (2 × CH_{Phth}), 144.9 (C_{Ts}), 168.5 (2 × C=O) ppm. FT-IR (ATR): $\tilde{\nu}$ = 3464 (w), 2947 (w), 1771 (m), 1704 (vs), 1597 (w), 1437 (m), 1396 (s), 1355 (s), 1188 (m), 1173 (s), 1097 (m), 1046 (m), 1018 (m), 935 (m), 904 (m), 814 (m), 717 (vs), 661 (s), 576 (m), 553 (s), 529 (m) cm^{-1}. MS (ESI) *m*/*z* = 396 $[M + Na]^+$, 374 $[M + H]^+$, 202 $[M–OTs]^+$. HRMS (ESI): calcd. for $[C_{19}H_{19}NO_5SNa]^+$ 396.0876, found: 396.0899 $[M + Na]^+$. Isotopic labeled **6a** is described in [10] and ^{1}H-NMR can be found in [11].

6-(1,3-Dioxo-1,3-dihydro-2H-isoindol-2-yl)hexyl 4-methylbenzenesulfonate (6b)

According to GP 4, from alcohol **5b** (3.47 g, 10.0 mmol), NEt_3 (3.00 mL, 2.23 g, 22.0 mmol), *p*-TsCl (2.48 g, 13.0 mmol), CH_2Cl_2 (35 mL), chromatography with hexanes/EtOAc (5:1), yield: 3.64 g, 9.06 mmol, 91%, orange oil; R_f = 0.28 (hexanes/EtOAc 5:1). ^{1}H-NMR (500 MHz, $CDCl_3$): δ = 1.21–1.40 (m, 4H, CH_2), 1.57–1.69 (m, 4H, CH_2), 2.45 (s, 3H, CH_3), 3.64 (t, J = 7.2 Hz, 2H, 1-H), 4.01 (t, J = 6.4 Hz, 2H, 6-H), 7.34 (d, J = 8.0 Hz, 2H, CH_{Ts}), 7.67–7.74 (m, 2H, CH_{Phth}), 7.78 (d, J = 8.0 Hz, 2H, CH_{Ts}), 7.81–7.86 (m, 2H, CH_{Phth}) ppm. ^{13}C-NMR (125 MHz, $CDCl_3$): δ = 21.7 (CH_3), 25.0, 26.2, 28.4, 28.7 (CH_2), 37.8 (C-1), 70.6 (C-6), 123.2 (2 × CH_{Phth}), 127.9 (2 × CH_{Ts}), 129.9 (2 × CH_{Ts}), 132.1 (2 × C_{Phth}), 133.2 (C_{Ts}), 134.0 (2 × CH_{Phth}), 144.7 (C_{Ts}), 168.5 (2 × C=O) ppm. FT-IR (ATR): $\tilde{\nu}$ = 2939 (w), 2862 (w), 1772 (w), 1710 (s), 1598 (w), 1467 (w), 1437 (w), 1397 (m), 1358 (m), 1188 (w), 1176 (w), 962 (w), 924 (w), 816 (w), 721 (m), 664 (m), 576 (w), 555 (m), 530 (w) cm^{-1}. MS (ESI) *m*/*z* = 424 $[M + Na]^+$, 402 $[M + H]^+$. HRMS (ESI): calcd. for $[C_{21}H_{23}NO_5SNa]^+$ 424.1189, found: 424.1172 $[M + Na]^+$. Product **6b** was partly described in [12].

8-(1,3-Dioxo-1,3-dihydro-2H-isoindol-2-yl)octyl 4-methylbenzenesulfonate (6c)

According to GP 4, from alcohol **5c** (3.08 g, 11.2 mmol), NEt_3 (3.40 mL, 2.49 g, 24.6 mmol), *p*-TsCl (2.78 g, 14.6 mmol), CH_2Cl_2 (40 mL), chromatography with hexanes/EtOAc (8:1), yield: 4.25 g, 9.89 mmol, 88%, light yellow oil, R_f = 0.53 (hexanes/EtOAc 8:1). ^{1}H-NMR (500 MHz, $CDCl_3$): δ = 1.16–1.37 (m, 8H, CH_2), 1.58–1.69 (m, 4H, CH_2), 2.45 (s, 3H, CH_3), 3.65 (t, J = 7.3 Hz, 2H, 1-H), 4.01 (t, J = 6.5 Hz, 2H, 8-H), 7.34 (d, J = 8.1 Hz, 2H, CH_{Ts}), 7.69–7.73 (m, 2H, CH_{Phth}), 7.79 (d, J = 8.1 Hz, 2H, CH_{Ts}), 7.81–7.86 (m, 2H, CH_{Phth}) ppm. ^{13}C-NMR (125 MHz, $CDCl_3$): δ = 21.6 (CH_3), 25.3, 26.7, 28.5, 28.7, 28.8, 28.9 (CH_2), 38.0 (C-1), 70.6 (C-8), 123.2 (2 × CH_{Phth}), 127.9 (2 × CH_{Ts}), 129.8 (2 × CH_{Ts}), 132.2 (2 × C_{Phth}), 133.3 (C_{Ts}), 133.9 (2 × CH_{Phth}), 144.7 (C_{Ts}), 168.5 (2 × C=O) ppm. FT-IR (ATR): $\tilde{\nu}$ = 2930 (w), 2857 (w), 1771 (w), 1706 (s), 1598 (w), 1467 (w), 1437 (w), 1395 (m), 1356 (m), 1307 (w), 1291 (w), 1188 (m), 1175 (s), 1097 (w), 1060 (w), 1020 (w), 942 (m), 816 (w), 792 (w), 719 (s), 690 (w), 664 (m), 622 (w), 576 (m), 554 (m), 530 (w) cm^{-1}. MS (ESI) *m*/*z* = 452 $[M + Na]^+$, 430 $[M + H]^+$, 362, 226, 209, 158. HRMS (ESI): calcd. for $[C_{23}H_{27}NO_5SNa]^+$ 452.1502, found: 452.1533 $[M + Na]^+$. Product **6c** was partly described in [13].

10-(1,3-Dioxo-1,3-dihydro-2H-isoindol-2-yl)decyl 4-methylbenzenesulfonate (6d)

According to GP 4, from alcohol **5d** (1.82 g, 6.00 mmol), NEt_3 (1.83 mL, 1.34 g, 13.2 mmol), *p*-TsCl (1.49 g, 7.80 mmol), CH_2Cl_2 (20 mL), chromatography with hexanes/EtOAc (8:1), yield: 2.45 g, 5.54 mmol, 92%, yellow oil; R_f = 0.23 (hexanes/EtOAc 8:1). ^{1}H-NMR (500 MHz, $CDCl_3$): δ = 1.15–1.35 (m, 12H, CH_2), 1.56–1.70 (m, 4H, CH_2), 2.45 (s, 3H, CH_3), 3.67 (t, J = 7.3 Hz, 2H, 1-H), 4.01 (t, J = 6.5 Hz, 2H, 10-H), 7.34 (d, J = 8.1 Hz, 2H, CH_{Ts}), 7.68–7.73 (m, 2H, CH_{Phth}), 7.79 (d, J = 8.1 Hz, 2H, CH_{Ts}), 7.82–7.86 (m, 2H, CH_{Phth}) ppm. ^{13}C-NMR (125 MHz, $CDCl_3$): δ = 21.7 (CH_3), 25.3, 26.8, 28.6, 28.8, 28.9, 29.1, 29.2, 29.3 (CH_2), 38.2 (C-1), 70.8 (C-10), 123.2 (2 × CH_{Phth}), 127.9 (2 × CH_{Ts}), 129.8 (2 × CH_{Ts}), 132.2

(2 × *C*Phth), 133.3 (*C*Ts), 133.8 (2 × *C*HPhth), 144.6 (*C*Ts), 168.5 (2 × C=O) ppm. FT-IR (ATR): $\tilde{\nu}$ = 2927 (m), 2855 (w), 1771 (w), 1710 (s), 1598 (w), 1495 (w), 1467 (w), 1437 (w), 1396 (m), 1359 (m), 1188 (m), 1176 (m), 1097 (w), 1046 (w), 1020 (w), 960 (m), 930 (m), 816 (w), 720 (w), 690 (w), 664 (m), 622 (w), 576 (w), 555 (m), 530 (w) cm^{-1}. MS (ESI) *m*/*z* = 480 $[M + Na]^+$, 458 $[M + H]^+$, 286. HRMS (ESI): calcd. for $[C_{25}H_{31}NO_5SNa]^+$ 480.1815, found: 480.1803 $[M + Na]^+$. Product **6d** was partly described in [13].

2-(4-Azidobutyl)-1H-isoindole-1,3(2H)-dione (7a)

(a) According to GP 5a, from **6a** (2.41 g, 6.46 mmol), TBAI (0.24 g, 0.65 mmol), NaN_3 (0.46 g, 7.1 mmol), DMF (40 mL), chromatography with hexanes/EtOAc (5:1), yield: 1.56 g, 6.37 mmol, 99%, colorless oil; R_f = 0.30 (hexanes/EtOAc 5:1).

(b) According to GP 5b, from **9a** (2.55 g, 9.05 mmol), NaN_3 (0.88 g, 13.6 mmol), DMF (50 mL), precipitation in ice water, yield: 2.08 g, 8.51 mmol, 94%, colorless solid. ^{1}H-NMR (300 MHz, $CDCl_3$): δ = 1.58–1.70 (m, 2H, CH_2), 1.71–1.84 (m, 2H, CH_2), 3.33 (t, *J* = 6.7 Hz, 2H, 4-H), 3.72 (t, *J* = 6.7 Hz, 2H, 1-H), 7.71 (dd, *J* = 5.4, 3.2 Hz, 2H, C*H*Phth), 7.84 (dd, *J* = 5.4, 3.2 Hz, 2H, C*H*Phth) ppm. ^{13}C-NMR (75 MHz, $CDCl_3$): δ = 26.0, 26.4 (C-2, C-3), 37.4 (C-1), 51.0 (C-4), 123.4 (2 × *C*HPhth), 132.2 (2 × *C*Phth), 134.2 (2 × *C*HPhth), 168.5 (2 × C=O) ppm. The spectroscopic data are in accordance with those in [14].

2-(6-Azidohexyl)-1H-isoindole-1,3(2H)-dione (7b)

(a) According to GP 5a, from **6b** (3.64 g, 9.06 mmol), TBAI (0.33 g, 0.91 mmol), NaN_3 (0.65 g, 9.97 mmol), DMF (100 mL), chromatography with hexanes/EtOAc (5:1), yield: 2.12 g, 7.78 mmol, 86%, yellow oil; R_f = 0.81 (hexanes/EtOAc 1:1).

(b) According to GP 5b, from **9b** (0.83 g, 2.68 mmol), NaN_3 (0.26 g, 4.01 mmol), DMF (15 mL), chromatography with hexanes/EtOAc (3:1), yield: 0.61 g, 2.23 mmol, 83%, colorless oil. ^{1}H-NMR (500 MHz, $CDCl_3$): δ = 1.33–1.46 (m, 4H, CH_2), 1.54–1.63 (m, 2H, 5-H), 1.65–1.74 (m, 2H, 2-H), 3.26 (t, *J* = 6.9 Hz, 2H, 6-H), 3.69 (t, *J* = 7.2 Hz, 2H, 1-H), 7.69–7.74 (m, 2H, C*H*Phth), 7.82–7.87 (m, 2H, C*H*Phth) ppm. ^{13}C-NMR (125 MHz, $CDCl_3$): δ = 26.3, 26.4 (CH_2), 28.5 (C-2), 28.7 (C-5), 37.8 (C-1), 51.3 (C-6), 123.2 (2 × *C*HPhth), 132.2 (2 × *C*Phth), 133.9 (2 × *C*HPhth), 168.7 (2 × C=O) ppm. Product **10b** is described in [15].

2-(8-Azidooctyl)-1H-isoindole-1,3(2H)-dione (7c)

(a) According to GP 5a, from **6c** (4.21 g, 9.80 mmol), TBAI (0.36 g, 0.98 mmol), NaN_3 (0.70 g, 10.8 mmol), DMF (80 mL), chromatography with hexanes/EtOAc (2:1), yield: 2.68 g, 8.92 mmol, 91%, yellow oil; R_f = 0.66 (hexanes/EtOAc 2:1).

(b) According to GP 5b, from **9c** (1.25 g, 3.70 mmol), NaN_3 (0.37 g, 5.69 mmol), DMF (25 mL), chromatography with hexanes/EtOAc (6:1), yield: 1.06 g, 3.53 mmol, 95%, colorless solid; M.p. 31 °C. ^{1}H-NMR (500 MHz, $CDCl_3$): δ = 1.28–1.39 (m, 8H, CH_2), 1.58 (tt, *J* = 7.0, 7.0 Hz, 2H, 7-H), 1.67 (tt, *J* = 7.1, 7.1 Hz, 2H, 2-H), 3.24 (t, *J* = 7.0 Hz, 2H, 8-H), 3.68 (t, *J* = 7.1 Hz, 2H, 1-H), 7.68–7.73 (m, 2H, C*H*Phth), 7.82–7.86 (m, 2H, C*H*Phth) ppm. ^{13}C-NMR (125 MHz, $CDCl_3$): δ = 26.6, 26.7 (CH_2), 28.5 (C-2), 28.8 (C-7), 28.97, 28.98 (CH_2), 38.2 (C-1), 51.7 (C-8), 123.3 (2 × *C*HPhth), 132.5 (2 × *C*Phth), 134.2 (2 × *C*HPhth), 168.7 (2 × C=O) ppm. FT-IR (ATR): $\tilde{\nu}$ = 2931 (w), 2857 (w), 2093 (m), 1773 (w), 1709 (s), 1615 (w), 1437 (w), 1396 (m), 1368 (m), 1257 (w), 1188 (w), 1156 (w), 1063 (w), 933 (w), 878 (w), 794 (w), 719 (m), 622 (w), 551 (w), 530 (w) cm^{-1}. MS (ESI) *m*/*z* = 323 $[M + Na]^+$, 301 $[M + H]^+$, 273, 250, 228. HRMS (ESI): calcd. for $[C_{16}H_{20}N_4O_2Na]^+$ 323.1478, found: 323.1486 $[M + Na]^+$. Product **7c** is partly described in literature [16].

2-(10-Azidodecyl)-1H-isoindole-1,3(2H)-dione (7d)

(a) According to GP 5a, from **6d** (2.54 g, 5.54 mmol), TBAI (0.20 g, 0.55 mmol), NaN_3 (0.40 g, 6.09 mmol), DMF (45 mL), chromatography with hexanes/EtOAc (1:1), yield: 1.38 g, 4.20 mmol, 76%, colorless oil; R_f = 0.83 (hexanes/EtOAc 1:1).

(b) According to GP 5b, from **9d** (3.60 g, 9.82 mmol), NaN_3 (0.98 g, 15.1 mmol), DMF (75 mL), chromatography with hexanes/EtOAc (6:1), yield: 2.87 g, 8.74 mmol, 89%, colorless solid. ^{1}H-NMR

(500 MHz, $CDCl_3$): δ = 1.24–1.34 (m, 12H, CH_2), 1.58 (tt, *J* = 7.0, 7.0 Hz, 2H, 9-H), 1.67 (tt, *J* = 7.2, 7.2 Hz, 2H, 2-H), 3.25 (t, *J* = 7.0 Hz, 2H, 10-H), 3.67 (t, *J* = 7.2 Hz, 2H, 1-H), 7.69–7.73 (m, 2H, CH_{Phth}), 7.82–7.86 (m, 2H, CH_{Phth}) ppm. ^{13}C-NMR (125 MHz, $CDCl_3$): δ = 26.7, 26.8 (CH_2), 28.6 (C-2), 28.8 (C-9), 29.08, 29.11, 29.33, 29.35 (CH_2), 38.2 (C-1), 51.7 (C-10), 123.2 (2 × CH_{Phth}), 132.2 (2 × C_{Phth}), 133.8 (2 × CH_{Phth}), 168.5 (2 × C=O) ppm. The spectroscopic data are in accordance with those in the literature [17].

2-[4-(Prop-2-ynyloxy)butyl]-1H-isoindole-1,3(2H)-dione (8a)

(a) According to GP 3, from alcohol **5a** (3.00 g, 13.7 mmol), NaH (60% in mineral oil, 0.55 g, 13.7 mmol), propargyl bromide (80% in toluene, 2.2 mL, 3.06 g, 20.6 mmol), DMF (35 mL), chromatography with hexanes/EtOAc (8:1, then 3:1 to 1:2), yield: **8a** 0.99 g, 3.83 mmol, 28%, orange solid, and **5a** 1.55 g, 7.07 mmol, 52%.

(b) Alternatively, from NaH (60% in mineral oil, 0.37 g, 9.28 mmol), propargyl alcohol (0.54 mL, 0.52 g, 9.28 mmol), bromide **9a** (2.38 g, 8.44 mmol), chromatography with hexanes/EtOAc (5:1), yield: 0.94 g, 3.65 mmol, 43%, colorless solid; R_f = 0.21 (hexanes/EtOAc 5:1), M.p. 54–56 °C. ^{1}H-NMR (300 MHz, $CDCl_3$): *δ* = 1.57–1.68 (m, 2H, 3-H), 1.70–1.82 (m, 2H, 2-H), 2.39 (t, *J* = 2.3 Hz, 1H, CC*H*), 3.53 (t, *J* = 6.2 Hz, 2H, 4-H), 3.70 (t, *J* = 6.9 Hz, 2H, 1-H), 4.10 (d, *J* = 2.3 Hz, 2H, OC*H*$_2$CCH), 7.70 (dd, *J* = 5.4, 3.1 Hz, 2H, CH_{Phth}), 7.82 (dd, *J* = 5.4, 3.1 Hz, 2H, CH_{Phth}) ppm. ^{13}C-NMR (75 MHz, $CDCl_3$): *δ* = 25.5 (C-2), 27.0 (C-3), 37.8 (C-1), 58.2 (OCH_2CCH), 69.5 (C-4), 74.3 (CCH), 80.0 (CCH), 123.3 (2 × CH_{Phth}), 132.3 (2 × C_{Phth}), 134.0 (2 × CH_{Phth}), 168.5 (2 × C=O) ppm. FT-IR (ATR): $\tilde{\nu}$ = 3303 (s), 2944 (s), 2866 (s), 2253 (s), 1771 (s), 1705 (s), 1396 (m), 1360 (m), 1092 (m), 1052 (m), 907 (m), 717 (s), 647 (m), 529 (m) cm^{-1}. MS (ESI): *m*/*z* = 280 [M + Na]$^+$, 202 [M-OCH_2CCH]$^+$, 160 [$C_9H_6NO_2$]$^+$. HRMS (ESI): calcd. for [$C_{15}H_{15}NO_3Na$]$^+$ 280.0944, found: 280.0966 [M + Na]$^+$. Product **8a** is mentioned in ref. [18].

2-[6-(Prop-2-ynyloxy)hexyl]-1H-isoindole-1,3(2H)-dione (8b)

(a) According to GP 3, from alcohol **5b** (5.00 g, 20.2 mmol), NaH (60% in mineral oil, 0.97 g, 24.2 mmol), propargyl bromide (80% in toluene, 2.70 mL, 3.60 g, 24.2 mmol), DMF (40 mL), chromatography with hexanes/EtOAc (8:1), yield: 1.23 g, 4.32 mmol, 21%, yellow oil. R_f = 0.28 (hexanes/EtOAc 5:1).

(b) Alternatively, from NaH (0.52 g, 13.0 mmol, 60% in mineral oil), propargyl alcohol (0.76 mL, 0.73 g, 13.0 mmol), bromide **9b** (3.52 g, 11.3 mmol), chromatography with hexanes/EtOAc (5:1), yield: 2.07 g, 7.25 mmol, 64%, colorless oil. ^{1}H-NMR (500 MHz, $CDCl_3$): *δ* = 1.32–1.46 (m, 4H, CH_2), 1.54–1.63 (m, 2H, 5-H), 1.64–1.73 (m, 2H, 2-H), 2.40 (t, *J* = 2.1 Hz, 1H, CC*H*), 3.50 (t, *J* = 6.3 Hz, 2H, 6-H), 3.68 (t, *J* = 7.3 Hz, 2H, 1-H), 4.11 (d, *J* = 2.1 Hz, 2H, OC*H*$_2$CCH), 7.69–7.73 (m, 2H, CH_{Phth}), 7.82–7.86 (m, 2H, CH_{Phth}) ppm. ^{13}C-NMR (125 MHz, $CDCl_3$): *δ* = 25.7 (C-3), 26.6 (C-4), 28.5 (C-2), 29.4 (C-5), 38.0 (C-1), 58.0 (OCH_2CCH), 70.0 (C-6), 74.1 (CCH), 80.0 (CCH), 123.2 (2 × CH_{Phth}), 132.2 (2 × C_{Phth}), 133.9 (2 × CH_{Phth}), 168.5 (2 × C=O) ppm. FT-IR (ATR): $\tilde{\nu}$ = 3273 (w), 2937 (w), 2860 (w), 1772 (w), 1709 (s), 1467 (w), 1438 (w), 1397 (m), 1369 (m), 1188 (w), 1099 (m), 1062 (m), 720 (m), 530 (w) cm^{-1}. MS (ESI) *m*/*z* = 308 [M + Na]$^+$, 241. HRMS (ESI): calcd. for [$C_{17}H_{19}NO_3Na$]$^+$ 308.1257, found: 308.1233 [M + Na]$^+$.

2-[8-(Prop-2-ynyloxy)octyl]-1H-isoindole-1,3(2H)-dione (8c)

(a) According to GP 3, from alcohol **5c** (9.72 g, 35.3 mmol), NaH (60% in mineral oil, 1.69 g, 42.4 mmol), propargyl bromide (80% in toluene, 4.72 mL, 6.30 g, 42.4 mmol), DMF (115 mL), chromatography with hexanes/EtOAc (8:1), yield: 1.83 g, 5.84 mmol, 17%, yellow oil; R_f = 0.36 (hexanes/EtOAc 8:1).

(b) Alternatively, from NaH (0.20 g, 5.09 mmol, 60% in mineral oil), propargyl alcohol (0.29 mL, 0.29 g, 5.09 mmol), bromide **9c** (1.50 g, 4.43 mmol), chromatography with hexanes/EtOAc (10:1 to 7:1 to 5:1), yield: 0.88 g, 2.81 mmol, 63%, colorless solid. M.p. 30 °C. ^{1}H-NMR (500 MHz, $CDCl_3$): *δ* = 1.28–1.38 (m, 8H, CH_2), 1.53–1.61 (m, 2H, 7-H), 1.62–1.71 (m, 2H, 2-H), 2.41 (t, *J* = 2.4 Hz, 1H, CC*H*), 3.50 (t, *J* = 6.6 Hz, 2H, 8-H), 3.67 (t, *J* = 7.3 Hz, 2H, 1-H), 4.12 (d, *J* = 2.4 Hz, 2H, OC*H*$_2$CCH), 7.68–7.73 (m, 2H, CH_{Phth}), 7.82–7.86 (m, 2H, CH_{Phth}) ppm. ^{13}C-NMR (125 MHz, $CDCl_3$): *δ* = 26.0, 26.8 (CH_2), 28.6 (C-2), 29.3 (C-7), 29.1, 29.4 (CH_2), 38.0 (C-1), 58.0 (OCH_2CCH), 70.2 (C-8), 74.1 (CCH),

80.1 (*C*CH), 123.2 (2 × *C*HPhth), 132.2 (2 × *C*Phth), 133.9 (2 × *C*HPhth), 168.5 (2 × C=O) ppm. FT-IR (ATR): $\tilde{\nu}$ = 3270 (w), 2931 (w), 2856 (w), 1772 (w), 1710 (s), 1615 (w), 1467 (w), 1438 (w), 1396 (m), 1368 (m), 1188 (w), 1100 (m), 942 (w), 795 (w), 720 (m), 530 (w) cm^{-1}. MS (ESI) *m*/*z* = 336 $[M + Na]^+$, 314 $[M + H]^+$, 258, 160. HRMS (ESI): calcd. for $[C_{19}H_{23}NO_3Na]^+$ 336.1570, found: 336.1546 $[M + Na]^+$.

2-[10-(Prop-2-ynyloxy)decyl]-1H-isoindole-1,3(2H)-dione (8d)

(a) According to GP 3, from alcohol **5d** (7.01 g, 23.1 mmol), NaH (60% in mineral oil, 1.11 g, 27.7 mmol), propargyl bromide (80% in toluene, 3.10 mL, 4.12 g, 24.7 mmol), DMF (60 mL), chromatography with hexanes/EtOAc (8:1), yield: 1.30 g, 3.81 mmol, 16%, orange oil; R_f = 0.50 (hexanes/EtOAc 8:1).

(b) Alternatively, from NaH (0.68 g, 16.9 mmol, 60% in mineral oil), propargyl alcohol (0.98 mL, 0.95 g, 16.9 mmol), bromide **9d** (5.40 g, 14.7 mmol), chromatography with hexanes/EtOAc (10:1 to 7:1), yield: 2.25 g, 6.58 mmol, 45%, colorless solid, M.p. 39 °C. ^{1}H-NMR (500 MHz, $CDCl_3$): *δ* = 1.22–1.38 (m, 12H, CH_2), 1.54–1.65 (m, 2H, 9-H), 1.67 (tt, *J* = 7.3, 7.1 Hz, 2H, 2-H), 2.41 (t, *J* = 2.3 Hz, 1H, CC*H*), 3.49 (t, *J* = 6.6 Hz, 2H, 10-H), 3.67 (t, *J* = 7.3 Hz, 2H, 1-H), 4.12 (d, *J* = 2.3 Hz, 2H, OCH_2CCH), 7.68–7.73 (m, 2H, C*H*Phth), 7.82–7.86 (m, 2H, C*H*Phth) ppm. ^{13}C-NMR (125 MHz, $CDCl_3$): *δ* = 26.1, 26.9 (CH_2), 28.6 (C-2), 29.3 (C-9), 29.49, 29.53, 29.59, 29.61 (CH_2), 38.1 (C-1), 58.0 (OCH_2CCH), 70.3 (C-10), 74.0 (CCH), 80.1 (*C*CH), 123.2 (2 × *C*HPhth), 132.2 (2 × *C*Phth), 133.9 (2 × *C*HPhth), 168.5 (2 × C=O) ppm. FT-IR (ATR): $\tilde{\nu}$ = 3271 (w), 2926 (m), 2854 (m), 1772 (w), 1709 (s), 1615 (w), 1467 (w), 1438 (w), 1396 (m), 1367 (m), 1188 (w), 1101 (m), 794 (w), 720 (m), 530 (w) cm^{-1}. MS (ESI) *m*/*z* = 364 $[M + Na]^+$, 342 $[M + H]^+$, 301, 286. HRMS (ESI): calcd. for $[C_{21}H_{27}NO_3Na]^+$ 364.1883, found: 364.1864 $[M + Na]^+$.

1.5. Synthesis of Triazoles (11) and (12)

2-[4-(4-{[4-(1,3-Dioxo-1,3-dihydro-2*H*-isoindol-2-yl)butoxy]methyl}-1*H*-1,2,3-triazol-1-yl)butyl]-1*H*-isoindole-1,3(2*H*)-dione (11a)

According to GP 6, from azide **7a** (1.15 g, 4.70 mmol), alkyne **8a** (1.21 g, 4.70 mmol), $CuSO_4 \cdot 5\ H_2O$ (12.0 mg, 0.05 mmol), sodium ascorbate (93.0 mg, 0.47 mmol), *t*-BuOH/H_2O (1:1, 30 mL), chromato-graphy with hexanes/EtOAc (1:1 to 1:2), yield: 1.85 g, 3.72 mmol, 79%, colorless solid, R_f = 0.19 (hexanes/EtOAc 1:2). M.p. 115–116 °C. ^{1}H-NMR (500 MHz, $CDCl_3$): *δ* = 1.58–1.64 (m, 2H, 3′′-H), 1.66–1.76 (m, 4H, 2′-H, 2′′-H), 1.90–1.97 (m, 2H, 3′-H), 3.51 (t, *J* = 6.4 Hz, 2H, 4′′-H), 3.65 (t, *J* = 7.2 Hz, 2H, 1′-H or 1′′-H), 3.70 (t, *J* = 6.9 Hz, 2H, 1′′-H or 1′-H), 4.39 (t, *J* = 7.2 Hz, 2H, 4′-H), 4.57 (s, 2H, 6-H), 7.58 (s, 1H, 5-H), 7.65–7.70 (m, 4H, 2 × C*H*Phth), 7.76 - 7.80 (m, 4H, 2 × C*H*Phth) ppm. ^{13}C-NMR (125 MHz, $CDCl_3$): *δ* = 25.4, 25.6 (C-2′, C-2′′), 26.9 (C-3′), 27.6 (C-3′′), 36.9, 37.8 (C-1′, C-1′′), 49.6 (C-4′), 64.5 (C-6), 69.9 (C-4′′), 122.4 (C-5), 123.2, 123.3 (each 2 × *C*HPhth), 132.0, 132.2 (each 2 × *C*Phth), 134.0, 134.1 (each 2 × *C*HPhth), 145.5 (C-4), 168.37, 168.42 (each 2 × C=O) ppm. FT-IR (ATR): $\tilde{\nu}$ = 3464 (w), 2942 (w), 2865 (w), 1770 (m), 1702 (vs), 1437 (m), 1395 (s), 1360 (m), 1089 (m), 1041 (m), 913 (m), 716 (s), 529 (m) cm^{-1}. MS (ESI): *m*/*z* = 502 $[M + H]^+$, 524 $[M + Na]^+$, 540 $[M + K]^+$. HRMS (ESI): calcd. for $[C_{27}H_{27}N_5O_5Na]^+$ 524.1904, found: 524.1934 $[M + Na]^+$.

2-{6-(4-({[6-(1,3-Dioxo-1,3-dihydro-2*H*-isoindol-2-yl)hexyl]oxy}methyl)-1*H*-1,2,3-triazol-1-yl)-hexyl}-1*H*-isoindole-1,3(2*H*)-dione (11b)

According to GP 6, from azide **7b** (545 mg, 2.00 mmol), alkyne **8b** (571 mg, 2.00 mmol), $CuSO_4 \cdot 5\ H_2O$ (5.0 mg, 0.02 mmol), sodium ascorbate (40.0 mg, 0.20 mmol), *t*-BuOH/H_2O (1:1, 20 mL), chromatography with hexanes/EtOAc (1:1), yield: 950 mg, 1.70 mmol, 85%, colorless solid, R_f = 0.14 (hexanes/EtOAc 1:1). M.p. 90 °C. ^{1}H-NMR (500 MHz, $CDCl_3$): δ = 1.32–1.44 (m, 8H, CH_2), 1.55–1.63 (m, 2H, CH_2), 1.63–1.74 (m, 4H, CH_2), 1.86–1.95 (m, 2H, CH_2), 3.51 (t, *J* = 6.5 Hz, 2H, 6′′-H), 3.67 (t, *J* = 7.6 Hz, 4H, 1′-H, 1′′-H), 4.34 (t, *J* = 7.2 Hz, 2H, 6′-H), 4.61 (s, 2H, 6-H), 7.54 (s, 1H, 5-H), 7.68–7.73 (m, 4H, C*H*Phth), 7.80–7.86 (m, 4H, C*H*Phth) ppm. ^{13}C-NMR (125 MHz, $CDCl_3$): *δ* = 25.8, 26.0, 26.2, 26.7, 28.3, 28.6, 29.5, 30.2 (CH_2), 37.7, 38.0 (C-1′, C-1′′), 50.3 (C-6′), 64.5 (C-6), 70.7 (C-6′′), 122.3 (C-5), 123.2, 123.3 (each 2 × *C*HPhth), 132.1, 132.2 (each 2 × *C*Phth), 133.9, 134.0 (each 2 × *C*HPhth), 145.5

(C-4), 168.46, 168.48 (each 2 × C=O) ppm. FT-IR (ATR): $\tilde{\nu}$ = 2937 (w), 2860 (w), 1771 (w), 1709 (s), 1614 (w), 1466 (w), 1437 (w), 1397 (m), 1370 (w), 1219 (w), 1188 (w), 1093 (w), 1057 (w), 891 (w), 796 (w), 720 (m), 530 (w) cm^{-1}. MS (ESI) *m*/*z* = 580 $[M + Na]^+$, 558 $[M + H]^+$. HRMS (ESI): calcd. for $[C_{31}H_{35}N_5O_5Na]^+$ 580.2530, found: 580.2511 $[M + Na]^+$.

2-{8-(4-({[8-(1,3-Dioxo-1,3-dihydro-2H-isoindol-2-yl)octyl]oxy}methyl)-1H-1,2,3-triazol-1-yl)octyl}-1H-isoindole-1,3(2H)-dione (11c)

According to GP 6, from azide **7c** (2.34 g, 7.98 mmol), alkyne **8c** (2.50 g, 7.98 mmol), $CuSO_4 \cdot 5\ H_2O$ (20 mg, 0.08 mmol), sodium-ascorbate (0.16 g, 0.80 mmol), *t*-BuOH/H_2O (1:1, 80 mL), chromatography with hexanes/ EtOAc (1:1), yield: 3.90 g, 6.35 mmol, 80%, colorless solid, R_f = 0.23 (hexanes/EtOAc 1:1). M.p. 92 °C. ^{1}H-NMR (500 MHz, $CDCl_3$): δ = 1.23–1.37 (m, 16H, CH_2), 1.54–1.61 (m, 2H, CH_2), 1.62–1.70 (m, 4H, CH_2), 1.84–1.93 (m, 2H, CH_2), 3.49 (t, *J* = 6.7 Hz, 2H, 8″-H), 3.67 (t, *J* = 7.3 Hz, 4H, 1′-H, 1″-H), 4.32 (t, *J* = 7.3 Hz, 2H, 8′-H), 4.61 (s, 2H, 6-H), 7.54 (s, 1H, 5-H), 7.68–7.73 (m, 4H, CH_{Phth}), 7.80–7.86 (m, 4H, CH_{Phth}) ppm. ^{13}C-NMR (125 MHz, $CDCl_3$): δ = 26.0, 26.4, 26.7, 26.8, 28.5, 28.6, 28.8, 28.9, 29.1, 29.3, 29.6, 30.3 (CH_2), 37.9, 38.0 (C-1′, C-1″), 50.3 (C-8′), 64.4 (C-6), 70.9 (C-8″), 122.1 (C-5), 123.2, 123.3 (each 2 × CH_{Phth}), 132.1, 132.2 (each 2 × C_{Phth}), 133.9, 134.0 (each 2 × CH_{Phth}), 145.5 (C-4), 168.5 (4 × C=O) ppm. FT-IR (ATR): $\tilde{\nu}$ = 2931 (w), 2856 (w), 1772 (w), 1709 (s), 1614 (w), 1466 (w), 1437 (w), 1396 (m), 1368 (m), 1217 (w), 1188 (w), 1049 (w), 795 (w), 720 (m), 622 (w), 530 (w) cm^{-1}. MS (ESI) *m*/*z* = 636 $[M + Na]^+$, 614 $[M + H]^+$. HRMS (ESI): calcd. for $[C_{35}H_{43}N_5O_5Na]^+$ 636.3156, found: 636.3131 $[M + Na]^+$.

2-{10-(4-({[10-(1,3-Dioxo-1,3-dihydro-2H-isoindol-2-yl)decyl]oxy}methyl)-1H-1,2,3-triazol-1-yl)-decyl}-1H-isoindole-1,3(2H)-dione (11d)

According to GP 6, from azide **7d** (1.25 g, 3.80 mmol), alkyne **8d** (1.30 g, 3.80 mmol), $CuSO_4 \cdot 5\ H_2O$ (10 mg, 0.04 mmol), sodium ascorbate (75 mg, 0.38 mmol), *t*-BuOH/H_2O (1:1, 40 mL), chromatography with hexanes/ EtOAc (2:1), yield: 2.13 g, 3.18 mmol, 84%, colorless solid, R_f = 0.18 (hexanes/EtOAc 2:1). M.p. 96 °C. ^{1}H-NMR (500 MHz, $CDCl_3$): δ = 1.20–1.37 (m, 24H, CH_2), 1.53–1.61 (m, 2H, CH_2), 1.62–1.71 (m, 4H, CH_2), 1.84–1.93 (m, 2H, CH_2), 3.50 (t, *J* = 6.7 Hz, 2H, 10″-H), 3.67 (t, *J* = 7.3 Hz, 4H, 1′-H, 1″-H), 4.32 (t, *J* = 7.3 Hz, 2H, 10′-H), 4.62 (s, 2H, 6-H), 7.52 (s, 1H, 5-H), 7.67–7.75 (m, 4H, CH_{Phth}), 7.79–7.88 (m, 4H, CH_{Phth}) ppm. ^{13}C-NMR (125 MHz, $CDCl_3$): δ = 26.1, 26.5, 26.7, 26.8, 28.5, 28.6, 28.9, 29.1, 29.2, 29.25, 29.28, 29.4 (2C), 29.5, 29.7, 30.3 (CH_2), 38.0, 38.1 (C-1′, C-1″), 50.4 (C-10′), 64.5 (C-6), 70.9 (C-10″), 122.1 (C-5), 123.2 (4 × CH_{Phth}), 132.2 (4 × C_{Phth}), 133.83, 133.85 (each 2 × CH_{Phth}), 145.5 (C-4), 168.6 (4 × C=O) ppm. FT-IR (ATR): $\tilde{\nu}$ = 2927 (m), 2854 (w), 1771 (w), 1709 (s), 1614 (w), 1466 (w), 1437 (w), 1396 (m), 1368 (m), 1336 (w), 1218 (w), 1188 (w), 1089 (w), 1048 (w), 794 (w), 720 (m), 620 (w), 530 (w) cm^{-1}. MS (ESI) *m*/*z* = 692 $[M + Na]^+$, 670 $[M + H]^+$. HRMS (ESI): calcd. for $[C_{39}H_{51}N_5O_5Na]^+$ 692.3782, found: 692.3789 $[M + Na]^+$.

4-{4-[(4-Aminobutoxy)methyl]-1H-1,2,3-triazol-1-yl}butan-1-amine (12a)

According to GP 7, from **11a** (1.64 g, 3.27 mmol), $N_2H_4 \cdot H_2O$ (2.00 mL, 2.10 g, 32.7 mmol), EtOH (160 mL), yield: 0.74 g, 3.04 mmol, 93%, yellow oil (>90% by ^{1}H-NMR). ^{1}H-NMR (500 MHz, $CDCl_3$): *δ* = 1.33–1.42 (br s, 4H, 2 × NH_2), 1.42–1.51 (m, 4H, 2′-H, 2′′-H), 1.57–1.64 (m, 2H, 3′′-H), 1.90–1.97 (m, 2H, 3′-H), 2.67 (t, *J* = 7.0 Hz, 2H, 1′-H or 1′′-H), 2.71 (t, *J* = 7.0 Hz, 2H, 1′′-H or 1′-H), 3.51 (t, *J* = 6.6 Hz, 2H, 4′′-H), 4.34 (t, *J* = 7.2 Hz, 2H, 4′-H), 4.59 (s, 2H, 6-H), 7.51 (s, 1H, 5-H) ppm. ^{13}C-NMR (125 MHz, $CDCl_3$): *δ* = 27.1 (C-3′′), 27.8 (C-3′), 30.5 (2C, C-2′, C-2′′), 41.5, 42.1 (C-1′, C-1′′), 50.3 (C-4′), 64.5 (C-6), 70.7 (C-4′′), 122.2 (C-5), 145.6 (C-4) ppm.

6-(4-{[(6-Aminohexyl)oxy]methyl}-1H-1,2,3-triazol-1-yl)hexan-1-amine (12b)

According to GP 7, from **11b** (1.24 g, 2.22 mmol), $N_2H_4 \cdot H_2O$ (1.08 mL, 1.11 g, 22.2 mmol), EtOH (120 mL), yield: 600 mg, 2.02 mmol, 91%, yellow oil (>90% by ^{1}H-NMR). ^{1}H-NMR (500 MHz, $CDCl_3$): δ = 1.29–1.40 (m, 8H, CH_2), 1.40–1.48 (m, 4H, CH_2), 1.56–1.65 (m, 2H, CH_2), 1.65 (br s, 4H, NH_2), 1.87–1.95 (m, 2H, CH_2), 2.68 (t, *J* = 7.0 Hz, 4H, 1′-H, 1″-H), 3.52 (t, *J* = 6.6 Hz, 2H, 6″-H), 4.34

(t, *J* = 6.9 Hz, 2H, 6′-H), 4.62 (s, 2H, 6-H), 7.51 (s, 1H, 5-H) ppm. ^{13}C-NMR (125 MHz, $CDCl_3$): δ = 26.0, 26.3, 26.4, 26.7, 29.6, 30.3, 33.3, 33.6 (CH_2), 41.9, 42.1 (C-1′, C-1″), 50.3 (C-6′), 64.4 (C-6), 70.8 (C-6″), 122.1 (C-5), 145.5 (C-4) ppm.

8-(4-{[(8-Aminooctyl)oxy]methyl}-1H-1,2,3-triazol-1-yl)octan-1-amine (12c)

According to GP 7, from **11c** (2.00 g, 3.26 mmol), $N_2H_4{\cdot}H_2O$ (1.58 mL, 1.63 g, 32.6 mmol), EtOH (170 mL), yield: 1.10 g, 3.11 mmol, 95%, yellow oil (>85% by ^{1}H-NMR). ^{1}H-NMR (500 MHz, $CDCl_3$): δ = 1.22–1.35 (m, 16H, CH_2), 1.40–1.49 (m, 4H, CH_2), 1.53–1.62 (m, 2H, CH_2), 1.84–1.93 (m, 2H, CH_2), 2.33 (br s, 4H, NH_2), 2.70 (t, *J* = 7.0 Hz, 4H, 1′-H, 1″-H), 3.51 (t, *J* = 6.7 Hz, 2H, 8″-H), 4.32 (t, *J* = 7.3 Hz, 2H, 8′-H), 4.62 (s, 2H, 6-H), 7.51 (s, 1H, 5-H) ppm. ^{13}C-NMR (125 MHz, $CDCl_3$): δ = 26.1, 26.4, 26.7, 26.8, 29.0, 29.2, 29.4 (2C), 29.7, 30.3, 33.3, 33.4 (CH_2), 41.97, 42.02 (C-1′, C-1″), 50.4 (C-8′), 64.4 (C-6), 70.9 (C-8″), 122.1 (C-5), 145.6 (C-4) ppm.

10-(4-{[(10-Aminodecyl)oxy]methyl}-1H-1,2,3-triazol-1-yl)decan-1-amine (12d)

According to GP 7, from **11d** (2.00 g, 2.98 mmol), $N_2H_4{\cdot}H_2O$ (1.45 mL, 1.49 g, 29.8 mmol), EtOH (150 mL), yield: 1.16 g, 2.82 mmol, 95%, yellow oil (>80% by ^{1}H-NMR). ^{1}H-NMR (500 MHz, $CDCl_3$): δ = 1.22–1.35 (m, 24H, CH_2), 1.43–1.52 (m, 4H, CH_2), 1.54–1.62 (m, 2H, CH_2), 1.85–1.92 (m, 2H, CH_2), 2.73 (t, *J* = 7.2 Hz, 4H, 1′-H, 1″-H), 2.81 (br s, 4H, NH_2), 3.51 (t, *J* = 7.3 Hz, 2H, 10″-H), 4.34 (t, *J* = 7.2 Hz, 2H, 10′-H), 4.62 (s, 2H, 6-H), 7.52 (s, 1H, 5-H) ppm. ^{13}C-NMR (125 MHz, $CDCl_3$): δ = 26.1, 26.5, 26.76, 26.78, 28.9, 29.0, 29.3, 29.37, 29.39, 29.5, 29.7, 30.3, 32.5, 33.4 (CH_2), 41.7 (2C, C-1′, C-1″), 50.3 (C-10′), 64.4 (C-6), 70.8 (C-10″), 122.1 (C-5), 145.5 (C-4) ppm.

1.6. Synthesis of Cross-Linkers (2) and ***Me-(2)***$^{+}$ ***I***$^{-}$

1-[4-(4-{[4-(2,5-Dioxo-2,5-dihydro-1H-pyrrol-1-yl)butoxy]methyl}-1H-1,2,3-triazol-1-yl)butyl]-1H-pyrrole-2,5-dione (2a)

According to GP 8, from **12a** (0.69 g, 2.87 mmol), NEt_3 (0.96 mL, 0.70 g, 6.89 mmol), maleic anhydride (0.68 g, 6.89 mmol), EtOH (120 mL), Ac_2O (27 mL, 29.3 g, 0.29 mol), NaOAc (0.94 g, 6.89 mmol), chromatography with hexanes/EtOAc (1:1, 1:2, then pure EtOAc), yield: 0.21 g, 0.52 mmol, 18%, colorless solid, R_f = 0.51 (EtOAc). M.p. 61 °C. ^{1}H-NMR (500 MHz, $CDCl_3$): *δ* = 1.54–1.70 (m, 6H, 2′-H, 2′′-H, 3′′-H), 1.86–1.93 (m, 2H, 3′-H), 3.50–3.54 (m, 4H, 4′′-H, 1′-H or 1′′-H), 3.56 (t, *J* = 7.0 Hz, 2H, 1′′-H or 1′-H), 4.38 (t, *J* = 7.1 Hz, 2H, 4′-H), 4.60 (s, 2H, 6-H), 6.67 (s, 2H, $C\mathit{H}_{maleimide}$), 6.70 (s, 2H, $C\mathit{H}_{maleimide}$), 7.57 (s, 1H, 5-H) ppm. ^{13}C-NMR (125 MHz, $CDCl_3$): *δ* = 25.4, 25.6, 26.9 (C-2′, C-2′′, C-3′′), 27.5 (C-3′), 36.9, 37.7 (C-1′, C-1′′), 49.6 (C-4′), 64.6 (C-6), 70.0 (C-4′′), 122.4 (C-5), 134.2, 134.3 (each 2 × $CH_{maleimide}$), 145.6 (C-4), 170.8, 170.9 (each 2 × C=O) ppm. FT-IR (ATR): $\tilde{\nu}$ = 3458 (w), 3096 (w), 2943 (w), 2867 (w), 1700 (vs), 1443 (w), 1409 (m), 1370 (w), 1150 (w), 1100 (m), 1049 (w), 828 (m), 695 (m) cm^{-1}. MS (ESI): *m*/*z* = 424 $[M + Na]^+$. HRMS (ESI): calcd. for $[C_{19}H_{23}N_5O_5Na]^+$ 424.1591, found: 424.1582 $[M + Na]^+$.

1-{6-[4-({[6-(2,5-Dioxo-2,5-dihydro-1H-pyrrol-1-yl)hexyl]oxy}methyl)-1H-1,2,3-triazol-1-yl]hexyl}-1H-pyrrole-2,5-dione (2b)

According to GP 8, from **12b** (259 mg, 870 μmol), NEt_3 (1.34 mL, 1.01 g, 10 mmol), maleic anhydride (680 mg, 10 mmol), EtOH (15 mL), Ac_2O (15 mL), NaOAc (0.82 g, 10 mmol), chromatography with hexanes/ EtOAc (1:1, 1:2 then pure EtOAc), yield: 100 mg, 220 μmol, 25%, colorless solid, R_f = 0.06 (hexanes/EtOAc 1:1). M.p. 67 °C. ^{1}H-NMR (500 MHz, $CDCl_3$): δ = 1.22–1.42 (m, 8H, CH_2), 1.50–1.60 (m, 6H, CH_2), 1.86–1.94 (m, 2H, CH_2), 3.50 (t, *J* = 7.3 Hz, 6H, 1′-H, 1″H, 6″-H), 4.33 (t, *J* = 7.3 Hz, 2H, 6′-H), 4.61 (s, 2H, 6-H), 6.68, 6.69 (2 s, 4H, $C\mathit{H}_{maleimide}$), 7.54 (s, 1H, 5-H) ppm. ^{13}C-NMR (125 MHz, $CDCl_3$): δ = 25.7, 26.0, 26.1, 26.6, 28.3, 28.5, 29.5, 30.1 (CH_2), 37.6, 37.8 (C-1′, C-1″), 50.2 (C-6′), 64.4 (C-6), 70.6 (C-6″), 122.1 (C-5), 134.0, 134.1 (each 2 × $CH_{maleimide}$), 145.5 (C-4), 170.8, 170.9 (each 2 × C=O) ppm. FT-IR (ATR): $\tilde{\nu}$ = 3458 (w), 3096 (w), 2936 (w), 2860 (w), 1768 (w), 1698 (s), 1441 (m), 1408 (m), 1370 (m), 1338 (w), 1221 (w), 1148 (w), 1103 (m), 1049 (w), 828 (m), 695 (w)

cm^{-1}. MS (ESI) *m*/*z* = 480 [M + Na]$^+$, 458 [M + H]$^+$, 403. HRMS (ESI): calcd. for $[C_{23}H_{31}N_5O_5Na]^+$ 480.2217, found: 480.2252 [M + Na]$^+$.

1-{8-[4-({[8-(2,5-Dioxo-2,5-dihydro-1H-pyrrol-1-yl)octyl]oxy}methyl)-1H-1,2,3-triazol-1-yl]octyl}-1H-pyrrole-2,5-dione (2c)

According to GP 8, from **12c** (1.15 g, 3.26 mmol), NEt_3 (4.52 mL, 3.30 g, 32.6 mmol), maleic anhydride (680 mg, 10 mmol), EtOH (130 mL), Ac_2O (50 mL), NaOAc (2.67 g, 32.6 mmol), chromatography with hexanes/ EtOAc (1:1), yield: 500 mg, 970 µmol, 30%, colorless solid, R_f = 0.16 (hexanes/EtOAc 1:1). M.p. 79 °C. ^{1}H-NMR (500 MHz, $CDCl_3$): δ = 1.20–1.36 (m, 16H, CH_2), 1.50–1.62 (m, 6H, CH_2), 1.84–1.94 (m, 2H, CH_2), 3.48–3.53 (m, 6H, 1′-H, 1″-H, 8″-H), 4.33 (t, *J* = 7.3 Hz, 2H, 8′-H), 4.62 (s, 2H, 6-H), 6.681, 6.685 (2 s, 4H, $CH_{maleimide}$), 7.52 (s, 1H, 5-H) ppm. ^{13}C-NMR (125 MHz, $CDCl_3$): δ = 26.0, 26.4, 26.5, 26.7, 28.4, 28.5, 28.7, 28.79, 28.81, 29.0, 29.3, 29.6, 30.2 (CH_2), 37.8, 37.9 (C-1′, C-1″), 50.3 (C-8′), 64.4 (C-6), 70.9 (C-8″), 122.1 (C-5), 134.04, 134.05 (each 2 × $CH_{maleimide}$), 145.6 (C-4), 170.89, 170.90 (each 2 × C=O) ppm. FT-IR (ATR): $\tilde{\nu}$ = 3088 (w), 2913 (m), 2852 (m), 1695 (s), 1467 (w), 1455 (w), 1417 (m), 1374 (w), 1336 (w), 1261 (w), 1214 (w), 1187 (w), 1146 (w), 1122 (w), 1097 (w), 1055 (w), 980 (w), 923 (w), 837 (m), 786 (w), 726 (w), 697 (m), 627 (w) cm^{-1}. MS (ESI) *m*/*z* = 536 [M + Na]$^+$, 514 [M + H]$^+$. HRMS (ESI): calcd. for $[C_{27}H_{39}N_5O_5Na]^+$ 536.2843, found: 536.2879 [M + Na]$^+$.

1-{10-[4-({[10-(2,5-Dioxo-2,5-dihydro-1H-pyrrol-1-yl)decyl]oxy}methyl)-1H-1,2,3-triazol-1-yl]decyl}-1H-pyrrole-2,5-dione (2d)

According to GP 8, from **12d** (1.22 g, 2.98 mmol), NEt_3 (4.13 mL, 3.02 g, 29.8 mmol), maleic anhydride (2.03 g, 29.8 mmol), EtOH (120 mL), Ac_2O (50 mL), NaOAc (2.44 g, 29.8 mmol), chromatography with hexanes/ EtOAc (2:1 to 1:1), yield: 376 mg, 660 µmol, 22%, colorless solid, R_f = 0.33 (hexanes/EtOAc 1:1). M.p. 90 °C. ^{1}H-NMR (500 MHz, $CDCl_3$): δ = 1.20–1.35 (m, 24H, CH_2), 1.52–1.61 (m, 6H, CH_2), 1.85–1.93 (m, 2H, CH_2), 3.50 (t, *J* = 7.3 Hz, 4H, 1′-H, 1″-H), 3.51 (t, *J* = 6.5 Hz, 2H, 10″-H), 4.33 (t, *J* = 7.6 Hz, 2H, 10′-H), 4.62 (s, 2H, 6-H), 6.68 (s, 4H, $CH_{maleimide}$), 7.51 (s, 1H, 5-H) ppm. ^{13}C-NMR (125 MHz, $CDCl_3$): δ = 26.1, 26.5, 26.6, 26.7, 28.4, 28.5, 28.9, 29.0, 29.1, 29.2, 29.3, 29.4, 29.5, 29.7, 30.3 (CH_2), 37.88. 37.93 (C-1′, C-1″), 50.4 (C-10′), 64.4 (C-6), 70.9 (C-10″), 122.1 (C-5), 134.0 (4 × $CH_{maleimide}$), 145.5 (C-4), 170.9 (4 × C=O) ppm. FT-IR (ATR): $\tilde{\nu}$ = 3088 (w), 2915 (m), 2848 (m), 1697 (s), 1467 (w), 1420 (w), 1374 (w), 1338 (w), 1216 (w), 1184 (w), 1122 (w), 1099 (w), 1054 (w), 837 (w), 786 (w), 723 (w), 698 (m), 627 (w) cm^{-1}. MS (ESI) *m*/*z* = 592 [M + Na]$^+$, 570 [M + H]$^+$. HRMS (ESI): calcd. for $[C_{31}H_{47}N_5O_5Na]^+$ 592.3469, found: 592.3487 [M + Na]$^+$.

4-{[4-(2,5-Dioxo-2,5-dihydro-1H-pyrrol-1-yl)butoxy]methyl}-1-[4-(2,5-dioxo-2,5-dihydro-1H-pyrrol-1-yl)butyl]-3-methyl-1H-1,2,3-triazol-3-ium iodide (Me-(2a)$^+$ I^-)

According to GP 9, from **2a** (43 mg, 0.107 mmol), MeI (0.13 mL, 0.30 g, 2.14 mmol), MeCN (1.1 mL), 8 d at 40 °C, yield: 54 mg, 0.099 mmol, 93%, yellow oil. ^{1}H-NMR (500 MHz, $CDCl_3$): δ = 1.55–1.68 (m, 4H, 2′′-H, 3′′-H), 1.69–1.76 (m, 2H, 2′-H), 2.02–2.10 (m, 2H, 3′-H), 3.50 (t, *J* = 7.0 Hz, 2H, 1″-H), 3.57 (t, *J* = 6.8 Hz, 2H, 1′-H), 3.64 (t, *J* = 5.9 Hz, 2H, 4″-H), 4.36 (s, 3H, CH_3), 4.79 (t, *J* = 7.3 Hz, 2H, 4′-H), 4.92 (s, 2H, 6-H), 6.69, 6.71 (2 s, 4H, $CH_{maleimide}$), 9.38 (s, 1H, 5-H) ppm. ^{13}C-NMR (125 MHz, $CDCl_3$): δ = 25.2, 25.3 (C-2′, C-2′′), 26.6, 26.7 (C-3′, C-3′′), 36.6 (C-1′), 37.5 (C-1″), 39.5 (CH_3), 53.6 (C-4′), 61.0 (C-6), 71.3 (C-4″), 130.9 (C-5), 134.3, 134.4 (each 2 × $CH_{maleimide}$), 140.8 (C-4), 170.91, 170.94 (each 2 × C=O) ppm. FT-IR (ATR): $\tilde{\nu}$ = 3455 (w), 3051 (w), 2944 (w), 2870 (w), 2187 (w), 1697 (vs), 1441 (m), 1407 (m), 1365 (m), 1261 (w), 1149 (w), 1103 (m), 915 (m), 825 (m), 724 (s), 693 (s), 641 (m) cm^{-1}. MS (ESI) *m*/*z* = 416 [M]$^+$. HRMS (ESI): calcd. for $[C_{20}H_{26}N_5O_5]^+$ 416.1928, found: 416.1925 [M]$^+$.

1-[6-(2,5-Dioxo-2,5-dihydro-1H-pyrrol-1-yl)hexyl]-4-({[6-(2,5-dioxo-2,5-dihydro-1H-pyrrol-1-yl)-hexyl]oxy}methyl)-3-methyl-1H-1,2,3-triazol-3-ium iodide (**Me-(2b)$^+$ I^-**)

According to GP 9, from **2b** (72 mg, 160 mol), MeI (0.20 mL, 0.45 g, 3.15 mmol), MeCN (1.5 mL), 18 h at 82 °C, yield: 90 mg, 0.15 mmol, 94%, yellow oil. ^{1}H-NMR (500 MHz, $CDCl_3$): δ = 1.26–1.48

(m, 8H, CH_2), 1.55–1.63 (m, 6H, CH_2), 2.02–2.10 (m, 2H, CH_2), 3.50 (t, *J* = 7.1 Hz, 4H, 1′-H, 1″-H), 3.61 (t, *J* = 6.3 Hz, 2H, 6″-H), 4.37 (s, 3H, CH_3), 4.73 (t, *J* = 6.9 Hz, 2H, 6′-H), 4.90 (s, 2H, 6-H), 6.70, 6.71 (2 s, 4H, $CH_{maleimide}$), 9.45 (s, 1H, 5-H) ppm. ^{13}C-NMR (125 MHz, $CDCl_3$): δ = 25.4, 25.5, 25.8, 26.3, 28.0, 28.3, 29.2, 29.3 (CH_2), 37.3, 37.6 (C-1′, C-1″), 39.2 (CH_3), 54.3 (C-6′), 60.8 (C-6), 72.1 (C-6″), 130.8 (C-5), 134.1 (4 × $CH_{maleimide}$), 140.6 (C-4), 170.88, 170.91 (each 2 × C=O) ppm. FT-IR (ATR): $\tilde{\nu}$ = 3459 (w), 3051 (w), 2933 (w), 2859 (w), 1700 (s), 1441 (w), 1408 (m), 1370 (m), 1231 (w), 1149 (w), 1110 (w), 829 (m), 729 (w), 695 (m), 639 (w) cm^{-1}. MS (ESI) *m*/*z* = 472 [M]$^+$. HRMS (ESI): calcd. for $[C_{24}H_{34}N_5O_5]^+$ 472.2554, found: 472.2572 [M]$^+$.

1-[8-(2,5-Dioxo-2,5-dihydro-1H-pyrrol-1-yl)octyl]-4-({[8-(2,5-dioxo-2,5-dihydro-1H-pyrrol-1-yl)octyl]oxy}methyl)-3-methyl-1H-1,2,3-triazol-3-ium iodide (**Me-(2c)$^+$ I$^-$**)

According to GP 9, from **2c** (200 mg, 390 μmol), MeI (0.49 mL, 1.11 g, 7.80 mmol), MeCN (3 mL), 4 d at 82 °C, yield: 255 mg, 390 μmol, quant., yellow solid. M.p. 65 °C. ^{1}H-NMR (500 MHz, $CDCl_3$): δ = 1.23–1.43 (m, 16H, CH_2), 1.53–1.63 (m, 6H, CH_2), 2.02–2.09 (m, 2H, CH_2), 3.50 (t, *J* = 6.9 Hz, 4H, 1′-H, 1″-H), 3.61 (t, *J* = 6.5 Hz, 2H, 8″-H), 4.36 (s, 3H, CH_3), 4.70 (t, *J* = 7.5 Hz, 2H, 8′-H), 4.90 (s, 2H, 6-H), 6.70 (s, 4H, $CH_{maleimide}$), 9.42 (s, 1H, 5-H) ppm. ^{13}C-NMR (125 MHz, $CDCl_3$): δ = 25.8, 25.9, 26.4, 26.5, 28.3, 28.4, 28.5, 28.9, 29.1, 29.3 (CH_2), 37.7, 37.8 (C-1′, C-1″), 39.1 (CH_3), 54.4 (C-8′), 60.8 (C-6), 72.3 (C-8″), 130.8 (C-5), 134.1 (4 × $CH_{maleimide}$), 140.6 (C-4), 170.9 (4 × C=O) ppm. FT-IR (ATR): $\tilde{\nu}$ = 3453 (w), 2931 (w), 2857 (w), 1703 (s), 1442 (w), 1408 (m), 1369 (w), 1112 (w), 831 (m), 695 (w) cm^{-1}. MS (ESI) *m*/*z* = 528 [M]$^+$. HRMS (ESI): calcd. for $[C_{28}H_{42}N_5O_5]^+$ 528.3180, found: 528.3200 [M]$^+$.

1-[10-(2,5-Dioxo-2,5-dihydro-1H-pyrrol-1-yl)decyl]-4-({[10-(2,5-dioxo-2,5-dihydro-1H-pyrrol-1-yl)decyl]oxy}methyl)-3-methyl-1H-1,2,3-triazol-3-ium iodide (**Me-(2d)$^+$ I$^-$**)

According to GP 9, from **2d** (150 mg, 260 μmol), MeI (0.33 mL, 0.75 g, 5.26 mmol), MeCN (2 mL), 4 d at 82 °C, yield: 185 mg, 260 μmol, quant., yellow solid. M.p. 78 °C. ^{1}H-NMR (500 MHz, $CDCl_3$): δ = 1.21–1.43 (m, 24H, CH_2), 1.53–1.63 (m, 6H, CH_2), 2.02–2.09 (m, 2H, CH_2), 3.50 (t, *J* = 7.3 Hz, 4H, 1′-H, 1″-H), 3.61 (t, *J* = 6.5 Hz, 2H, 10″-H), 4.36 (s, 3H, CH_3), 4.45 (t, *J* = 7.5 Hz, 2H, 10′-H), 4.90 (s, 2H, 6-H), 6.69 (s, 4H, $CH_{maleimide}$), 9.39 (s, 1H, 5-H) ppm. ^{13}C-NMR (125 MHz, $CDCl_3$): δ = 26.0, 26.1, 26.6, 26.7, 28.4, 28.5, 28.7, 28.9, 29.0, 29.1, 29.27, 29.31, 29.35, 29.43, 29.44 (CH_2), 37.85, 37.89 (C-1′, C-1″), 39.2 (CH_3), 54.5 (C-10′), 60.8 (C-6), 72.4 (C-10″), 130.7 (C-5), 134.1 (4 × $CH_{maleimide}$), 140.6 (C-4), 170.9 (4 × C=O) ppm. FT-IR (ATR): $\tilde{\nu}$ = 3456 (w), 2927 (m), 2855 (w), 1702 (s), 1442 (w), 1408 (m), 1369 (w), 1113 (w), 830 (m), 695 (m) cm^{-1}. MS (ESI) *m*/*z* = 616, 584 [M]$^+$. HRMS (ESI): calcd. for $[C_{32}H_{50}N_5O_5]^+$ 584.3806, found: 584.3851 [M]$^+$.

1.6. Thio-Michael Addition Products (13)

Methyl ({1-[6-(4-{[(6-{3-[(2-methoxy-2-oxoethyl)thio]-2,5-dioxopyrrolidin-1-yl}hexyl)oxy]methyl}-1H-1,2,3-triazol-1-yl)hexyl]-2,5-dioxopyrrolidin-3-yl}thio)acetate (13a)

According to GP 10, from **2b** (36 mg, 79 μmol), methyl thioglycolate (18 μL, 21 mg, 0.20 mmol), EtOH (3.0 mL), PBS (pH = 3, 3.0 mL), conversion: 100%, isolated yield: 47 mg, 70 μmol, 88%, colorless oil. ^{1}H-NMR (500 MHz, $CDCl_3$): δ = 1.23–1.36 (m, 8H, CH_2), 1.49–1.57 (m, 6H, CH_2), 1.82–1.89 (m, 2H, CH_2), 2.45, 2.46 (2 dd, *J* = 18.8, 3.7 Hz, 2 × 1H, 8′-H_a, 8′′-H_a), 3.095, 3.103 (2 dd, *J* = 18.8, 9.0 Hz, 2 × 1H, 8′-H_b, 8′′-H_b), 3.34, 3.35 (2 d, *J* = 15.8 Hz, 2 × 1H, 9′-H_a, 9′′-H_a), 3.42–3.48 (m, 6H, 1′-H, 1′′-H, 6′′-H), 3.71 (s, 6H, 2 × OCH_3), 3.855, 3.861 (2 d, *J* = 15.8 Hz, 2 × 1H, 9′-H_b, 9′′-H_b), 3.973, 3.977 (2 dd, *J* = 9.0, 3.7 Hz, 2 × 1H, 7′-H, 7′′-H), 4.29 (t, *J* = 7.2 Hz, 2H, 6′-H), 4.56 (s, 2H, 6-H), 7.50 (s, 1H, 5-H) ppm. ^{13}C-NMR (125 MHz, $CDCl_3$): δ = 25.7, 26.0, 26.1, 26.5, 27.2, 27.4, 29.4, 30.0 (8 × CH_2), 32.89, 32.91 (C-9′, C-9′′), 35.35, 35.36 (C-8′, C-8′′), 38.3 (2 C, C-7′, C-7′′), 38.8, 39.0 (C-1′, C-1″), 50.1 (C-6′), 52.7 (2 × OCH_3), 64.3 (C-6), 70.6 (C-6″), 122.2 (C-5), 145.4 (C-4), 170.06, 170.08 (2 × CO_2CH_3), 174.4, 176.4 (4 × C=O) ppm. FT-IR (ATR): $\tilde{\nu}$ = 2937 (w), 2860 (w), 1775 (w), 1734 (m), 1695 (s), 1436 (m), 1399 (m), 1370 (w), 1282 (w), 1160 (m), 1131 (m), 1050 (w), 1006 (w), 730 (w), 692 (w) cm^{-1}. MS (ESI) *m*/*z* = 692 [M + Na]$^+$. HRMS (ESI): calcd. for $[C_{29}H_{43}N_5O_9S_2Na]^+$ 692.2394, found: 692.2400 [M + Na]$^+$.

1-(6-{3-[(2-Methoxy-2-oxoethyl)thio]-2,5-dioxopyrrolidin-1-yl}hexyl)-4-{[(6-{3-[(2-methoxy-2-oxoethyl)thio]-2,5-dioxopyrrolidin-1-yl}hexyl)oxy]methyl}-3-methyl-1H-1,2,3-triazol-3-ium iodide (13b)

According to GP 10, from **Me-(2b)$^+$ I$^-$** (33 mg, 55 μmol), methyl thioglycolate (12 μL, 15 mg, 0.14 mmol), EtOH (1.5 mL), PBS (pH = 3, 1.5 mL), conversion: 100%, isolated yield: 38 mg, 47 μmol, 85%, yellow oil. ^{1}H-NMR (500 MHz, $CDCl_3$): δ = 1.23–1.35 (m, 6H, CH_2), 1.35–1.42 (m, 2H, CH_2), 1.49–1.59 (m, 6H, CH_2), 1.98–2.05 (m, 2H, 5′-H), 2.44, 2.45 (2 dd, *J* = 18.8, 3.8 Hz, 2 × 1H, 8′-H_a, 8′′-H_a), 3.14, 3.19 (2 dd, *J* = 18.8, 9.0 Hz, 2 × 1H, 8′-H_b, 8′′-H_b), 3.36, 3.37 (2 d, *J* = 15.7 Hz, 2 × 1H, 9′-H_a, 9′′-H_a), 3.41–3.46 (m, 4H, 1′-H, 1′′-H), 3.56 (t, *J* = 6.5 Hz, 2H, 6′′-H), 3.71 (s, 6H, 2 × OC*H*$_3$), 3.81, 3.83 (2 d, *J* = 15.7 Hz, 2 × 1H, 9′-H_b, 9′′-H_b), 4.02, 4.06 (2 dd, *J* = 9.0, 3.7 Hz, 2 × 1H, 7′-H, 7′′-H), 4.34 (s, 3H, CH_3), 4.69 (t, *J* = 7.2 Hz, 2H, 6′-H), 4.87 (s, 2H, 6-H), 9.36 (s, 1H, 5-H) ppm. ^{13}C-NMR (125 MHz, $CDCl_3$): δ = 25.4, 25.5, 25.7, 26.3, 27.0, 27.3, 29.16, 29.17, (8 × CH_2), 32.96, 33.00 (C-9′, C-9′′), 35.5, 35.6 (C-8′, C-8′′), 38.6, 38.7, 38.9 (4 C, C-1′, C-1′′, C-7′, C-7′′), 39.4 (CH_3), 52.75, 52.76 (2 × OCH_3), 54.2 (C-6′), 60.8 (C-6), 71.9 (C-6′′), 130.7 (C-5), 140.6 (C-4), 170.1 (2 × *C*O_2CH_3), 174.5, 174.6, 176.37, 176.44 (4 × C=O) ppm. FT-IR (ATR): $\tilde{\nu}$ = 2937 (w), 2859 (w), 1773 (w), 1732 (m), 1693 (s), 1436 (w), 1398 (m), 1347 (w), 1280 (w), 1160 (w), 1126 (w), 1005 (w), 918 (w), 728 (m), 690 (w) cm^{-1}. MS (ESI) *m*/*z* = 684 [M]$^+$. HRMS (ESI): calcd. for $[C_{30}H_{46}N_5O_9S_2Na]^+$ 684.2731, found: 684.2724 [M]$^+$.

1.7. Reaction Kinetics of the Thio-Michael Addition

Reaction kinetics of the thio-Michael addition were studied by NMR experiments using cross-linkers **2b** and **Me-(2b)$^+$ I$^-$** and methyl thioglycolate (MTG) in deuterated PBS/ethanol-d_6 (70% in D_2O) with a maleimide/thiol ratio of (50:50). Due to solubility problems a solvent ratio of PBS in D_2O (pH 3)/70% ethanol-d_6 of (50:50) was used instead of (70:30) as for the hydrogel formation.

Experimental Procedure

The cross-linkers **2b** (10.75 mg, 23.5 μmol) or **Me-(2b)$^+$ I$^-$** (14.09 mg, 23.5 μmol), ethanol-d_6 (70% in D_2O, 332 μL) and PBS in D_2O (pH 3, 357 μL) were mixed thoroughly in a standard NMR tube, and ^{1}H- and ^{13}C-NMR spectra were recorded. A stock solution of methyl thioglycolate (42 μL, 470 μmol) in ethanol-d_6 (70%, 250 μL, c = 199.5 mg/mL, 1.88 mmol/mL) was prepared.

Then, 25 μL of the stock solution of methyl thioglycolate (47.0 μmol) were added to the NMR tubes, the reaction mixture was mixed carefully, and ^{1}H-NMR spectra were recorded immediately. The reactions were monitored by ^{1}H-NMR until no further reaction progress could be detected. Afterwards, ^{13}C-NMR spectra were measured overnight.

The reaction mixture was extracted with CH_2Cl_2 (2 × 5 mL), the organic layer was separated and dried over Na_2SO_4. After removal of the solvent, ^{1}H-NMR spectra of the products in $CDCl_3$ were recorded.

Reaction Kinetics

(a) Reaction of Triazole Cross-Linker (2b) and Methyl Thioglycolate (MTG) in a Ratio of (1:2)

The neutral cross-linker **2b** did not completely dissolve in the D_2O/EtOH-d_6 mixture. After the addition of methyl thioglycolate, a cloudy suspension was formed. However, the reaction progress could still be monitored by ^{1}H-NMR and is shown in Figure S1.

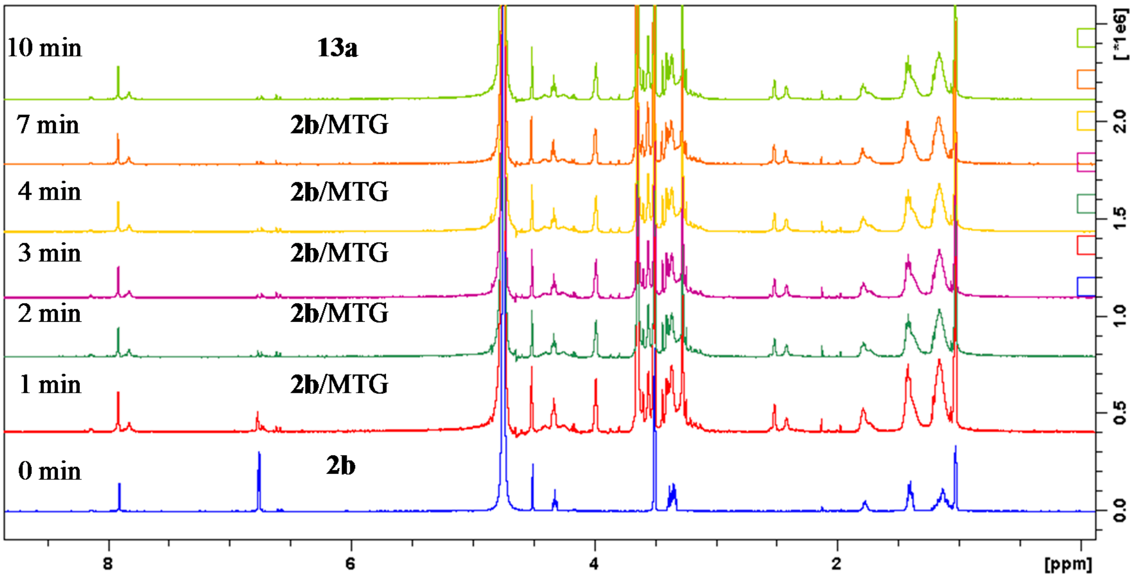

Figure S1. NMR experiment of the thio-Michael addition of MTG to neutral cross-linker **2b**.

The maleimide protons of the cross-linker at 6.80 ppm (Figure S1, blue spectrum, **2b**) disappeared within 10 min after the addition of methyl thioglycolate, due to the thio-Michael addition with MTG to **13a**, as can be seen by the appearance of CH_2 protons at 2.47 ppm and 4.05 ppm (red, green, and violet spectra). The reaction seemed to be finished after 3 min.

(b) Reaction of Triazolium Cross-Linker (**Me-(2b)$^+$ I$^-$**) and Methyl Thioglycolate (MTG) in a Ratio of (1:2)

The solubility of **Me-(2b)$^+$ I$^-$** in the D_2O/EtOH-d_6 mixture was higher than that of **2b**. After the addition of MTG, a colorless suspension was formed. The reaction progress of the thio-Michael addition was monitored by ^{1}H-NMR (Figure S2).

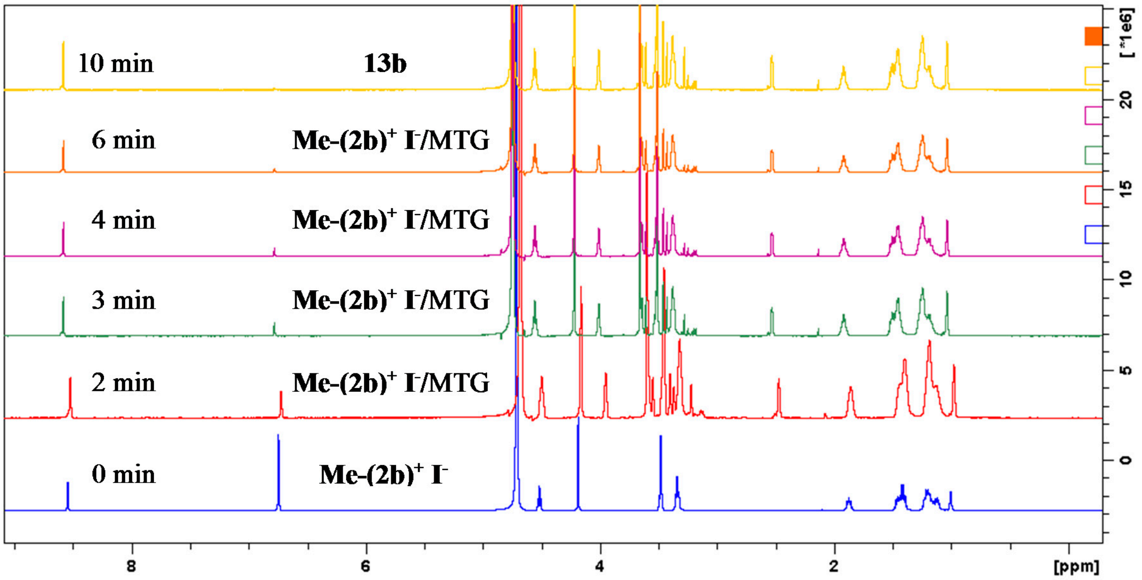

Figure S2. NMR experiment of the thio-Michael addition of MTG to positively charged cross-linker **Me-(2b)$^+$ I$^-$**.

After the addition of methyl thioglycolate, the maleimide protons at 6.80 ppm (Figure S2, blue spectrum, **Me-(2b)$^+$ I$^-$**) disappeared within 10 min due to the thio-Michael addition with MTG to **13b**, as can be seen by the appearance of CH_2 protons at 2.58 ppm and 4.05 ppm (red, green, and

violet spectra). The reaction was finished after 10 min reaction time. After work-up, no starting material **Me-(2b)$^+$ I$^-$** was detected by ^{1}H-NMR, only the desired thio-Michael adduct **13b**.

2. Formation of Hydrogels

2.1. Stability of HA_{125} and HA_{125}-SH_{40} in PBS at pH = 3.0

As hydrogels were prepared in PBS at a final pH of 3.0 with a gelation time of 24 h, stability of HA_{125} and HA_{125}-SH_{40} under these conditions was tested. Therefore HA_{125} and HA_{125}-SH_{40} were dissolved in PBS at a final pH = 3.0 and incubated for 24 h at room temperature. Subsequently, these HA solutions were loaded on a 1.4% agarose gel, together with fresh solutions of HA_{125} and HA_{125}-SH_{40} to determine possible degradation. Agarose gel electrophoresis was done in accordance with ref. [19].

Both HA_{125} and HA_{125}-SH_{40} have a broad size distribution but are not significantly degraded after incubation in PBS at pH = 3.0 for 24 h (Figure S3).

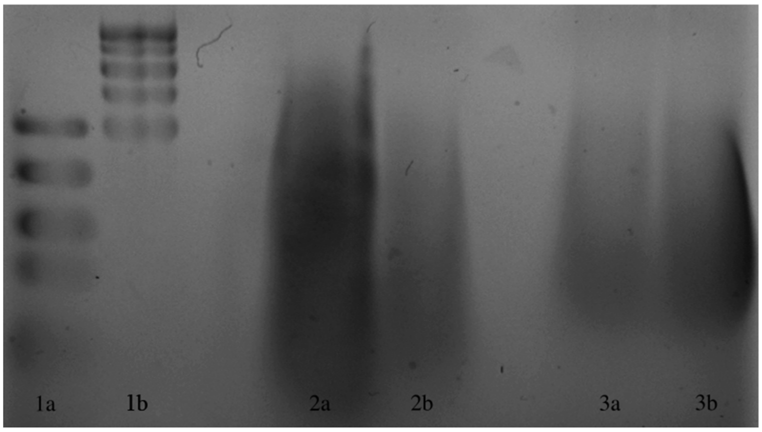

Figure S3. Agarose-gel of HA_{125} and HA_{125}-SH_{40} in PBS at a final pH = 3.0. **1a** is loaded with LoLadder (Hyalose) ranging from 500 kDa to 25 kDa and **1b** is loaded with HighLadder (Hyalose) ranging from 1500 kDa to 500 kDa. **2a** and **2b** represent HA_{125} freshly prepared and after incubation at pH = 3.0 for 24 h respectively. **3a** and **3b** show HA_{125}-SH_{40} freshly prepared and after pH = 3.0 incubation.

2.2. Determination of Reacted Thiols

An adapted Ellman's Assay (see Materials and Methods 3.2) was used to determine the amount of reacted thiols in hydrogels with all cross-linkers (Results shown in Table 1). In short, formed gels with a volume of 40 µL were immersed in 784 µL 1 M TRIS, pH = 8.0 and mechanically cut into small pieces. Subsequently 784 µL DTNB-solution (50 mM NaOAc and 2 mM DTNB in ddH_2O) were added, and the reaction mixture was incubated for 20 min at room temperature and 350 rpm. The amount of reacted thiols was calculated in reference to a 40 µL gelation solution without cross-linker, containing 100% free thiols.

2.3. Determination of Electrostatic Interactions within Hydrogels

Rheological measurements during gelation of HA_{125}-SH_{40} with **13a** and **13b**, containing protected maleimides, were performed with a rotational rheometer (Kinexus Pro, Malvern) at a constant shear rate of 1 Hz in a humidity chamber. Storage and loss modulus were monitored over a period of 24 h to determine possible gelation (Figure S4).

The uncharged protected linker **13a** showed no crossover of the two moduli, thus indicating no gel formation due to missing covalent linkages. However, with **13b** a crossover point is observed after roughly 19 h, indicating that there is a very weak gel formed (G′ ≈ 15 Pa after 24 h). The electrostatic interactions between the positive charge on the linker and the negative charge on the HA backbone are thus stabilizing the gel network even without covalent links.

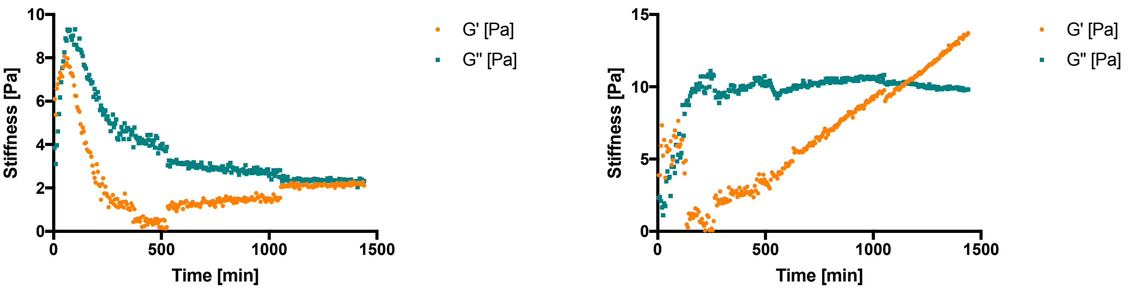

(**a**) (**b**)

Figure S4. Rheological measurements of HA_{125}-SH_{40} with **13a** (**a**) and **13b** (**b**) during gelation. Storage (green) and loss modulus (orange) were monitored over a time frame of 24 h to determine gelation. No crossover between storage and loss modulus can be obtained with **13a**, confirming that only covalent crosslinks are forming the hydrogels with uncharged linkers. However, with the charged crosslinker **13b** a gelation point can be observed after 19 h indicating electrostatic interactions.

3. References

1. Falck, J.R.; Wallukat, G.; Puli, N.; Goli, M.; Arnold, C.; Konkel, A.; Rothe, M.; Fischer, R.; Müller, D.N.; Schunck, W.-H. 17(*R*),18(*S*)-Epoxyeicosatetraenoic Acid, a Potent Eicosapentaenoic Acid (EPA) Derived Regulator of Cardiomyocyte Contraction: Structure–Activity Relationships and Stable Analogues. *J. Med. Chem.* **2011**, *54*, 4109–4118.
2. Kress, K.C.; Kaller, M.; Axenov, K.V.; Tussetschläger, S.; Laschat, S. Synthesis and mesomorphic properties of calamitic malonates and cyanoacetates tethered to 4-cyanobiphenyls. *Beilstein J. Org. Chem.* **2012**, *8*, 371–378.
3. Baldwin, J.E.; Adlington, R.M.; Ramcharitar, S.H. Free Radical Macrocyclisation *via* Propiolate Esters. *Tetrahedron* **1992**, *48*, 3413–3428.
4. Eriks, J.C.; van der Goot, H.; Sterk, G.J.; Timmerman, H. Histamine H2-Receptor Agonists. Synthesis, in Vitro Pharmacology, and Qualitative Structure-Activity Relationships of Substituted 4- and 5-(2-Aminoethyl)thiazoles. *J. Med. Chem.* **1992**, *35*, 3239–3246.
5. Hirschhäuser, C.; Velcicky, J.; Schlawe, D.; Hessler, E.; Majdalani, A.; Neudörfl, J.-M.; Prokop, A.; Wieder, T.; Schmalz, H.-G. Nucleoside Analogues with a 1,3-Diene-$Fe(CO)_3$ Substructure: Stereoselective Synthesis, Configurational Assignment, and Apoptosis-Inducing Activity. *Chem. Eur. J.* **2013**, *19*, 13017–13029.
6. Sugawara, T.; Susaki, H.; Nogusa, H.; Gonsho, A.; Iwasawa, H.; Irie, K.; Ito, Y.; Shibukawa, M. Synthesis of 8-aminooctyl glycopyranosides and of their conjugates with poly(L-glutamic acid) having a 2-(4-hydroxyphenyl)ethylamino group for radiolabeling. *Carbohyd. Res.* **1993**, *238*, 163–184.
7. Pascale, R.; Carocci, A.; Catalano, A.; Lentini, G.; Spagnoletta, A.; Cavalluzzi, M.M.; De Santis, F.; De Palma, A.; Scalera, V.; Franchini, C. New *N*-(phenoxydecyl)phthalimide derivatives displaying potent inhibition activity towards α-glucosidase. *Bioorg. Med. Chem.* **2010**, *18*, 5903–5914.
8. Kong, X.; He, Z.; Zhang, Y.; Mu, L.; Liang, C.; Chen, B.; Jing, X.; Cammidge, A.N. A Mesogenic Triphenylene-Perylene-Triphenylene Triad. *Org. Lett.* **2011**, *13*, 764-767.
9. Pfammatter, M.J.; Siljegovic, V.; Darbre, T.; Keese, R. Synthesis of ω-Substituted Alkanethiols and (Bromomethyl)methylthiomalonates. *Helv. Chim. Acta* **2001**, *84*, 678–689.
10. Thiruvengadam, T.K.; Gould, S.J.; Aberhart, D.J.; Lin, H.-J. Biosynthesis of Streptothricin F. 5. Formation of *ß*-Lysine by *Streptomyces* L-1689-23. *J. Am. Chem. Soc.* **1983**, *105*, 5470–5476.
11. Nagata, T.; Tanaka, K. Pentadentate Terpyridine−Catechol Linked Ligands and Their Cobalt(III) Complexes. *Inorg. Chem.* **2000**, *39*, 3515–3521.
12. Harjani, J.R.; Liang, C.; Jessop, P.G. A Synthesis of Acetamidines. *J. Org. Chem.* **2011**, *76*, 1683–1691.
13. Bartholomä, M.D.; Vortherms, A.R.; Hillier, S.; Ploier, B.; Joyal, J.; Babich, J.; Doyle, R.P.; Zubieta, J. Synthesis, Cytotoxicity, and Insight into the Mode of Action of $Re(CO)_3$ Thymidine Complexes. *ChemMedChem* **2010**, *5*, 1513–1529.
14. Lakanen, J.R.; Coward, J.K.; Pegg, A.E. α-Methyl Polyamines: Metabolically Stable Spermidine and Spermine Mimics Capable of Supporting Growth in Cells Depleted of Polyamines. *J. Med. Chem.* **1992**, *35*, 724–734.

15. Loska, R.; Janiga, A.; Gryko, D. Design and synthesis of protoporphyrin IX/vitamin B_{12} molecular hybrids *via* CuAAC reaction. *J. Porphyr. Phthalocya.* **2013**, *17*, 104–117.
16. Bandyopadhyay, S.; Mukherjee, S.; Dey, A. Modular synthesis, spectroscopic characterization and *in situ* functionalization using "click" chemistry of azide terminated amide containing self-assembled monolayers. *RSC Adv.* **2013**, *3*, 17174–17187.
17. Fer, M.J.; Bouhss, A.; Patrão, M.; Le Corre, L.; Pietrancosta, N.; Amoroso, A.; Joris, B.; Mengin-Lecreulx, D.; Calvet-Vitale, S.; Gravier-Pelletier, C. 5′-Methylene-triazole-substituted-aminoribosyl uridines as MraY inhibitors: Synthesis, biological evaluation and molecular modeling. *Org. Biomol. Chem.* **2015**, *13*, 7193–7222.
18. Talybov, G.M.; Guseinov, M.M.; Guseinov, S.O.; Karaev, S.F. Propargyl ether of 4-chlorobutanol and its derivatives. *Doklady Akademiya Nauk Azerbaidzhanskoi SSR* **1989**, *45*, 26–29.
19. Bhilocha, S.; Amin, R.; Pandya, M.; Yuan, H.; Tank, M.; LoBello, J.; Shytuhina, A.; Wang, W.; Wisniewski, H.-G.; de la Motte, C.; et al. Agarose and polyacrylamide gel electrophoresis methods for molecular mass analysis of 5- to 500-kDa hyaluronan. *Anal. Biochem.* **2011**, *417*, 41–49.

4. ^{1}H- and [13] C-NMR Spectra of All New Compounds

1-[4-(4-{[4-(2,5-Dioxo-2,5-dihydro-1*H*-pyrrol-1-yl)butoxy]methyl}-1*H*-1,2,3-triazol-1-yl)butyl]-1*H*-pyrrole-2,5-dione (2a)

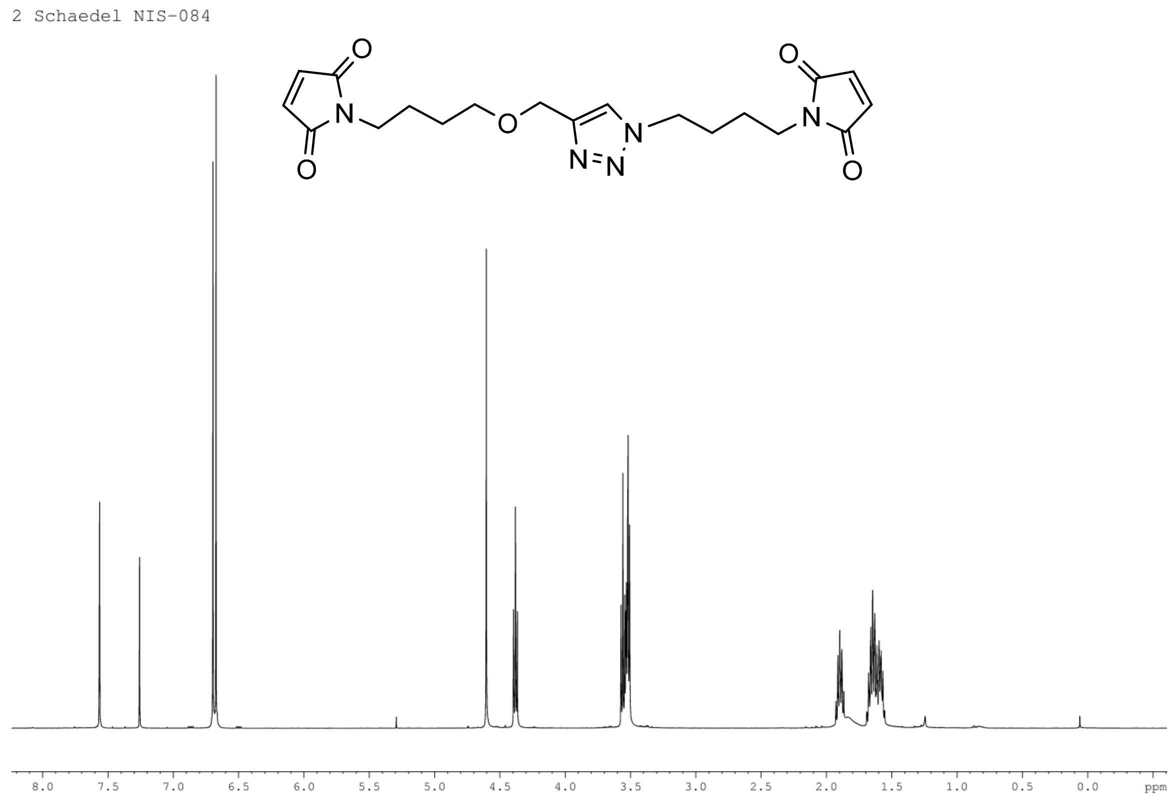

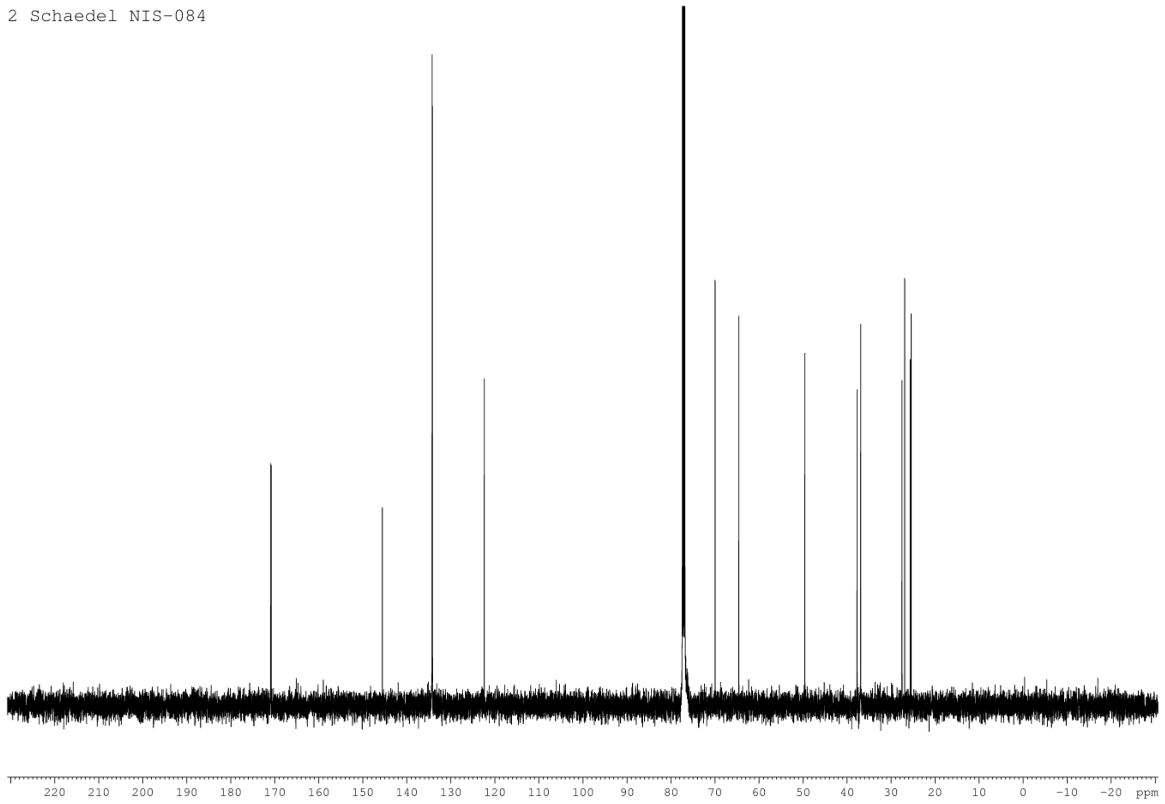

1-{6-[4-({[6-(2,5-Dioxo-2,5-dihydro-1H-pyrrol-1-yl)hexyl]oxy}methyl)-1H-1,2,3-triazol-1-yl]hexyl}-1H-pyrrole-2,5-dione (2b)

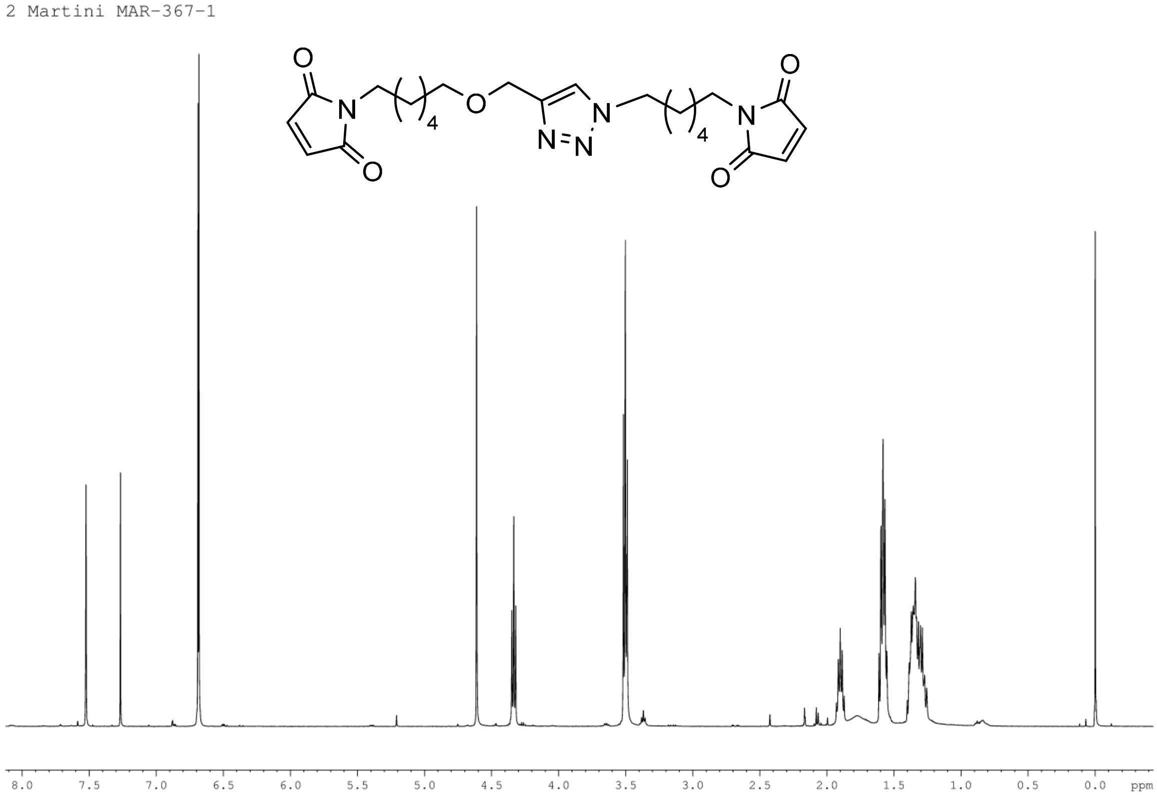

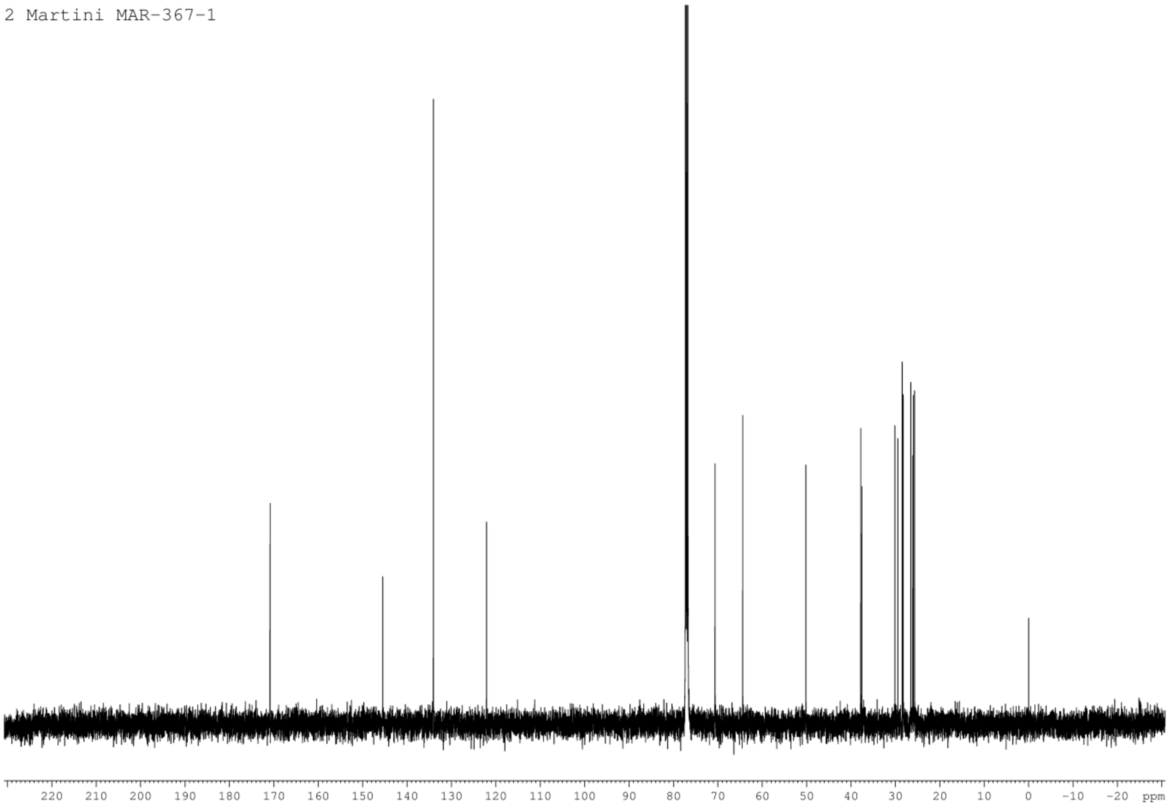

1-{8-[4-({[8-(2,5-Dioxo-2,5-dihydro-1H-pyrrol-1-yl)octyl]oxy}methyl)-1H-1,2,3-triazol-1-yl]octyl}-1H-pyrrole-2,5-dione (2c)

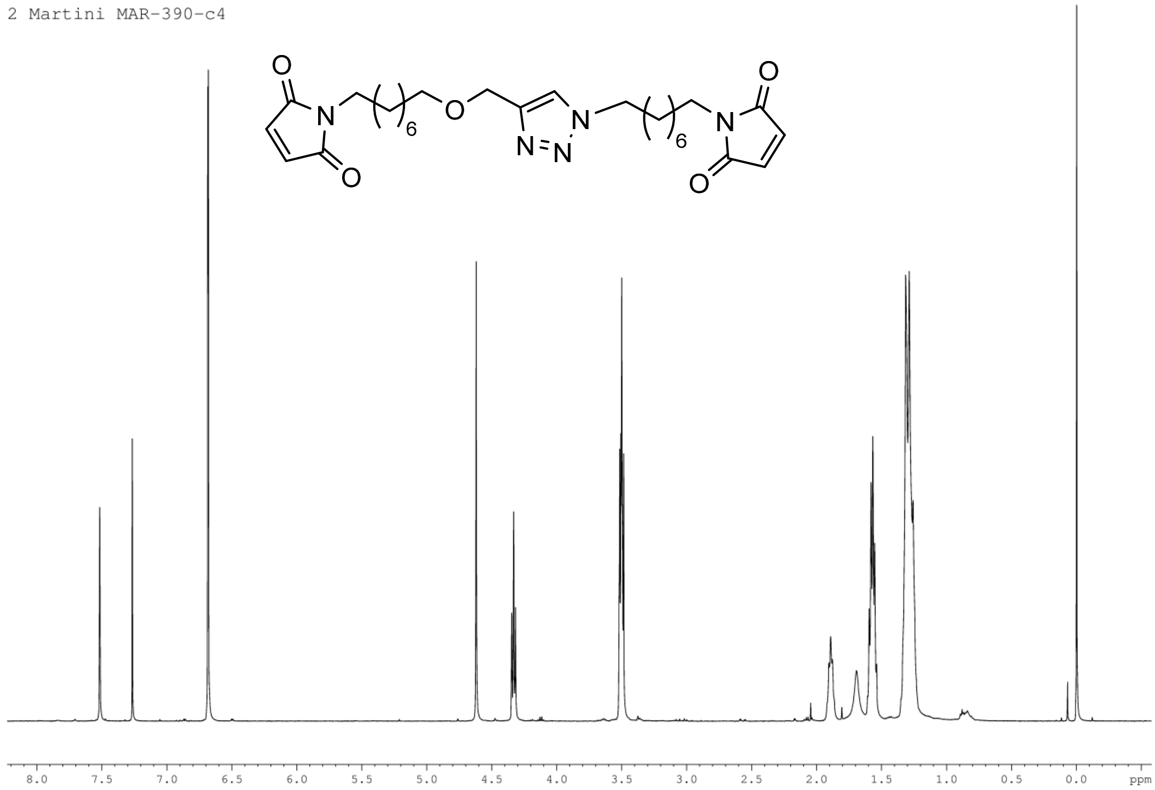

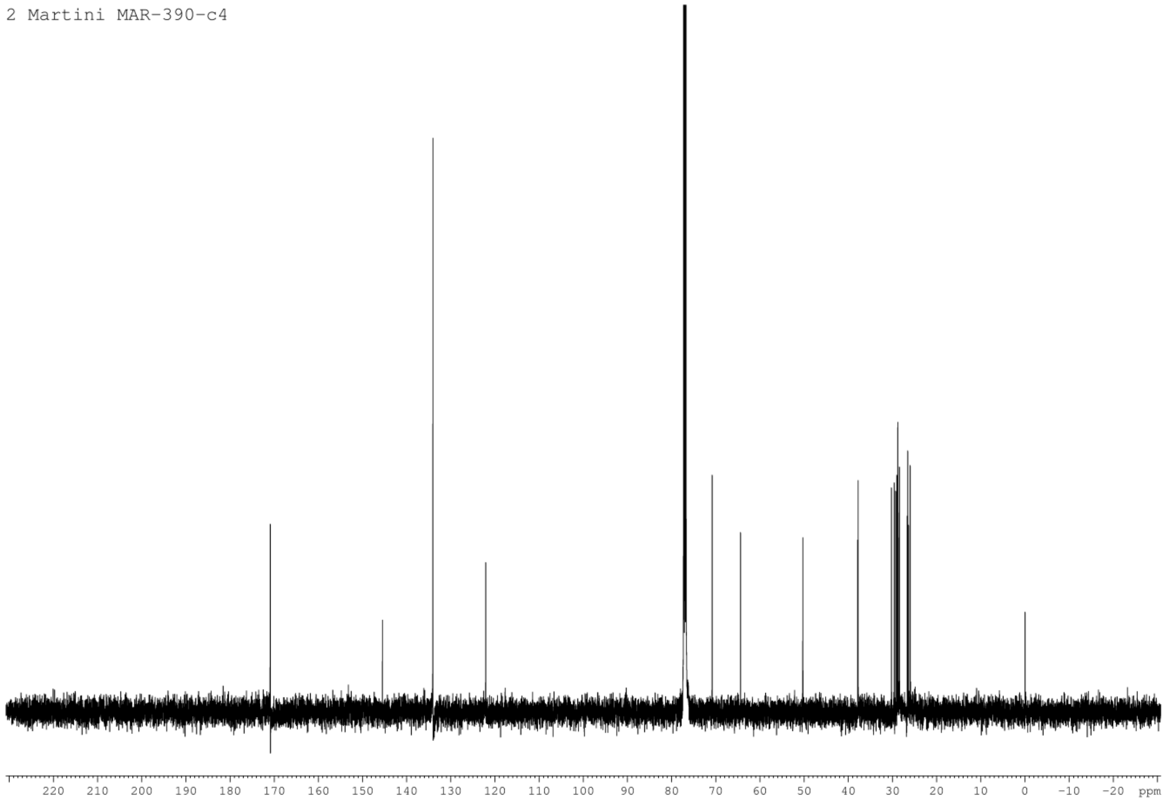

1-{10-[4-({[10-(2,5-Dioxo-2,5-dihydro-1H-pyrrol-1-yl)decyl]oxy}methyl)-1H-1,2,3-triazol-1-yl]decyl}-1H-pyrrole-2,5-dione (2d)

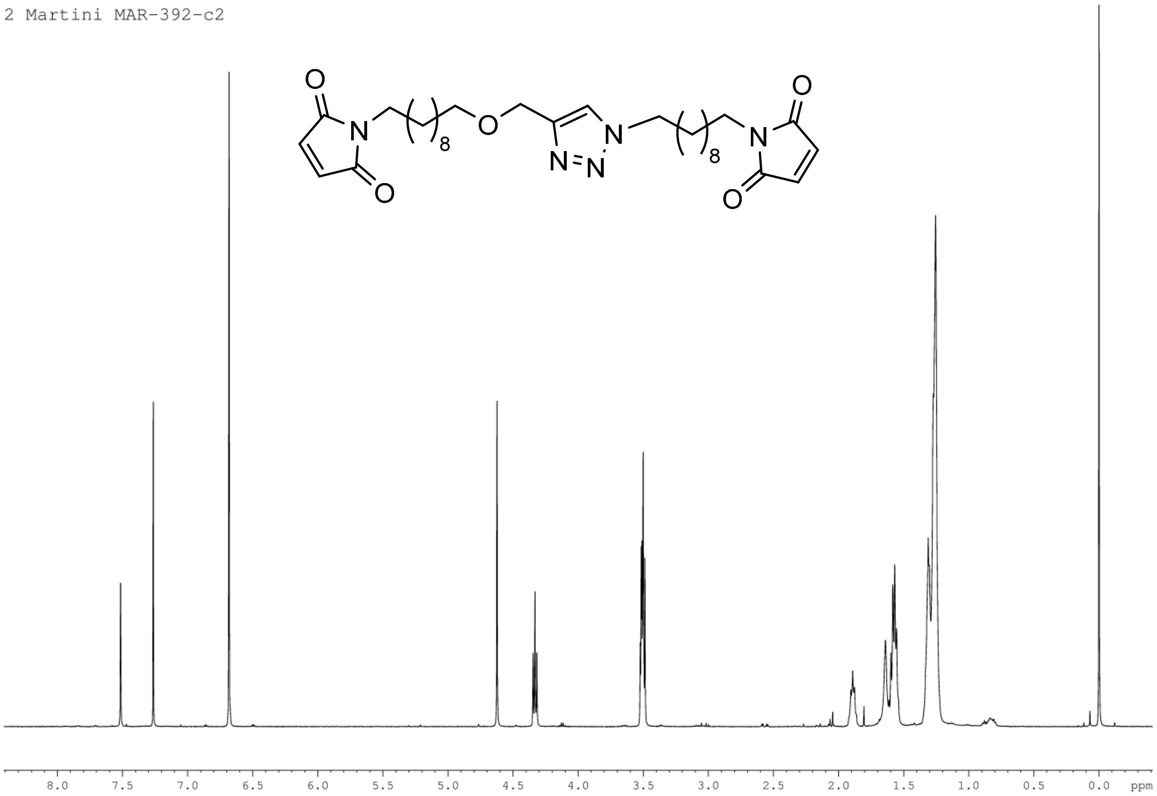

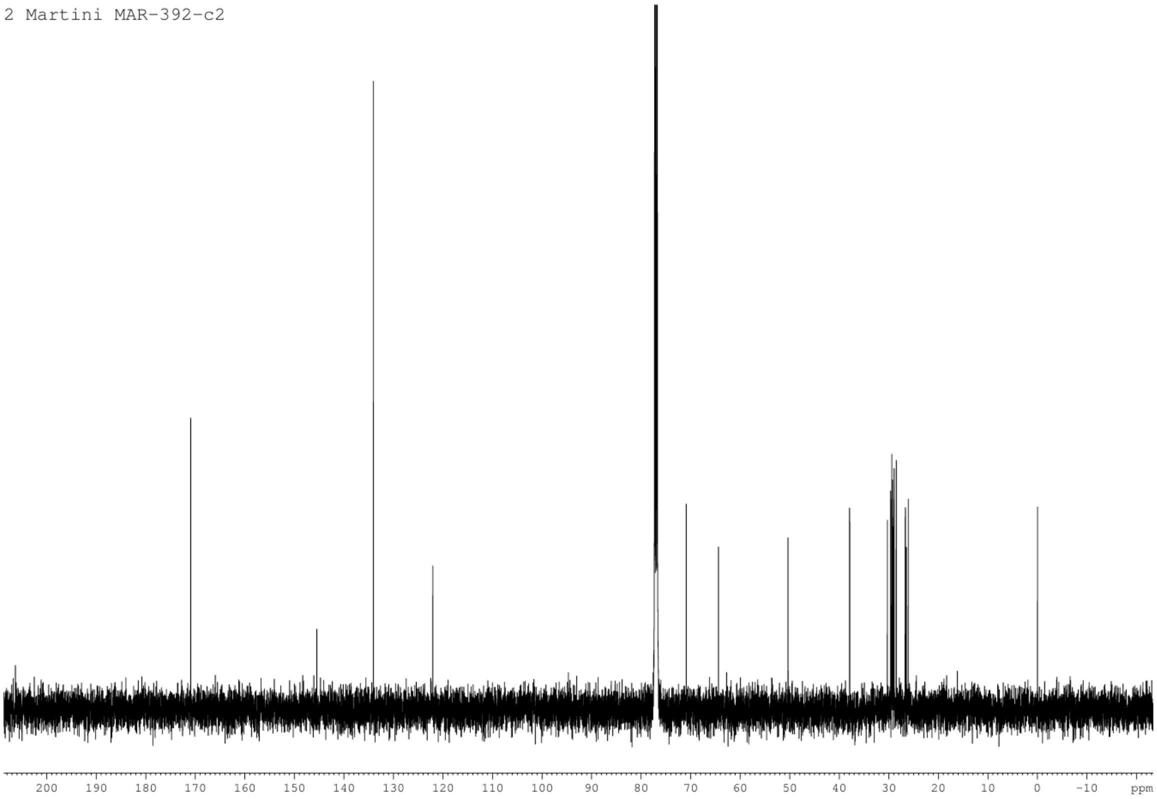

4-{[4-(2,5-Dioxo-2,5-dihydro-1H-pyrrol-1-yl)butoxy]methyl}-1-[4-(2,5-dioxo-2,5-dihydro-1H-pyrrol-1-yl)butyl]-3-methyl-1H-1,2,3-triazol-3-ium iodide (Me-(2a)+ I−)

2 Schaedel NIS091

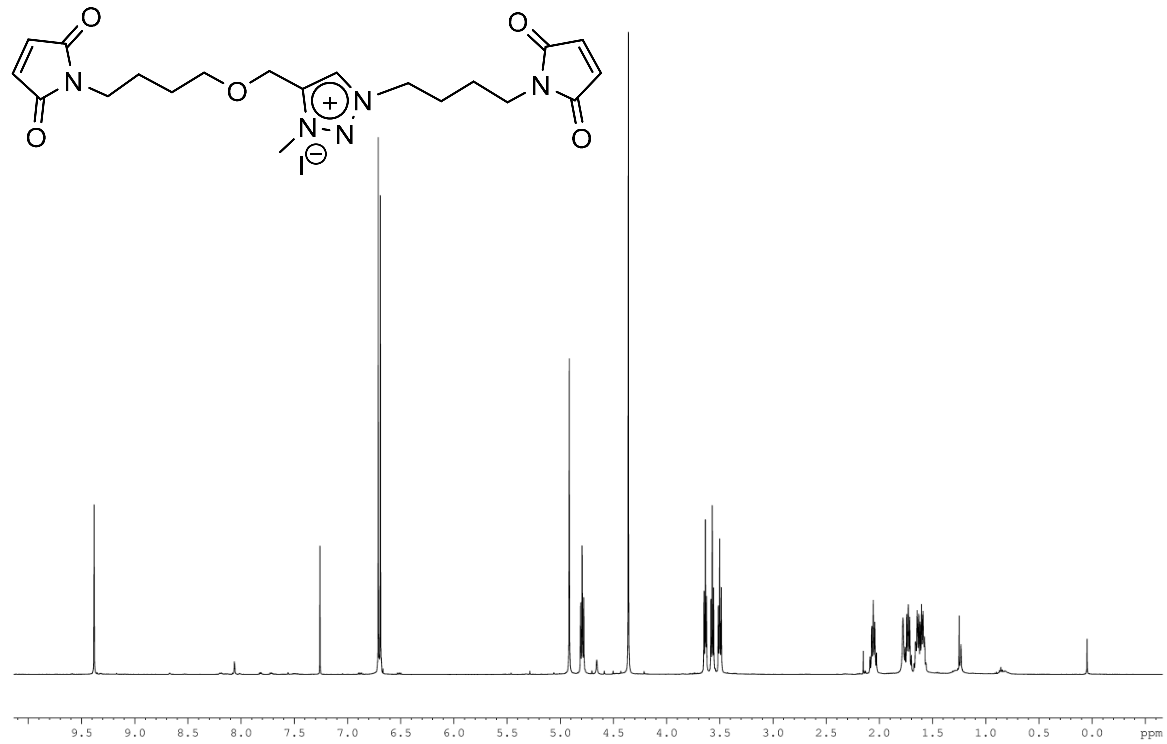

2 Schaedel NIS091

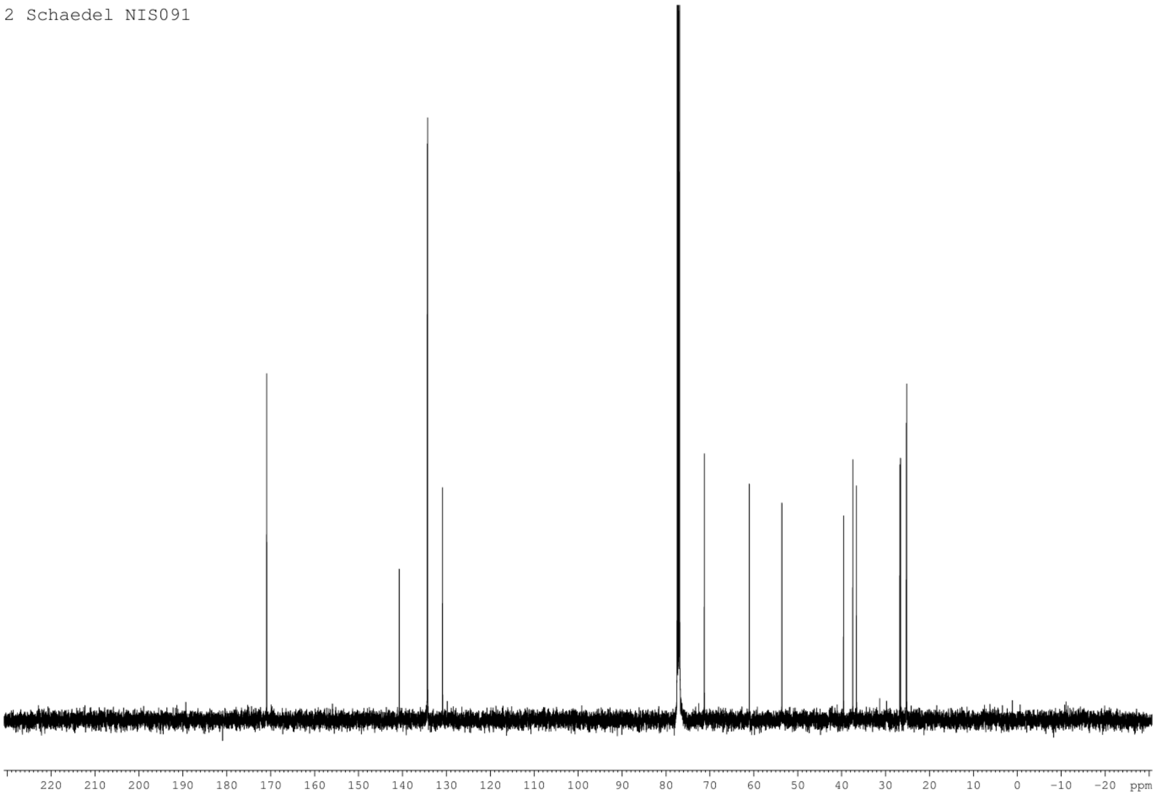

1-[6-(2,5-Dioxo-2,5-dihydro-1H-pyrrol-1-yl)hexyl]-4-({[6-(2,5-dioxo-2,5-dihydro-1H-pyrrol-1-yl)-hexyl]oxy}methyl)-3-methyl-1H-1,2,3-triazol-3-ium iodide (Me-(2b)$^+$ I$^-$)

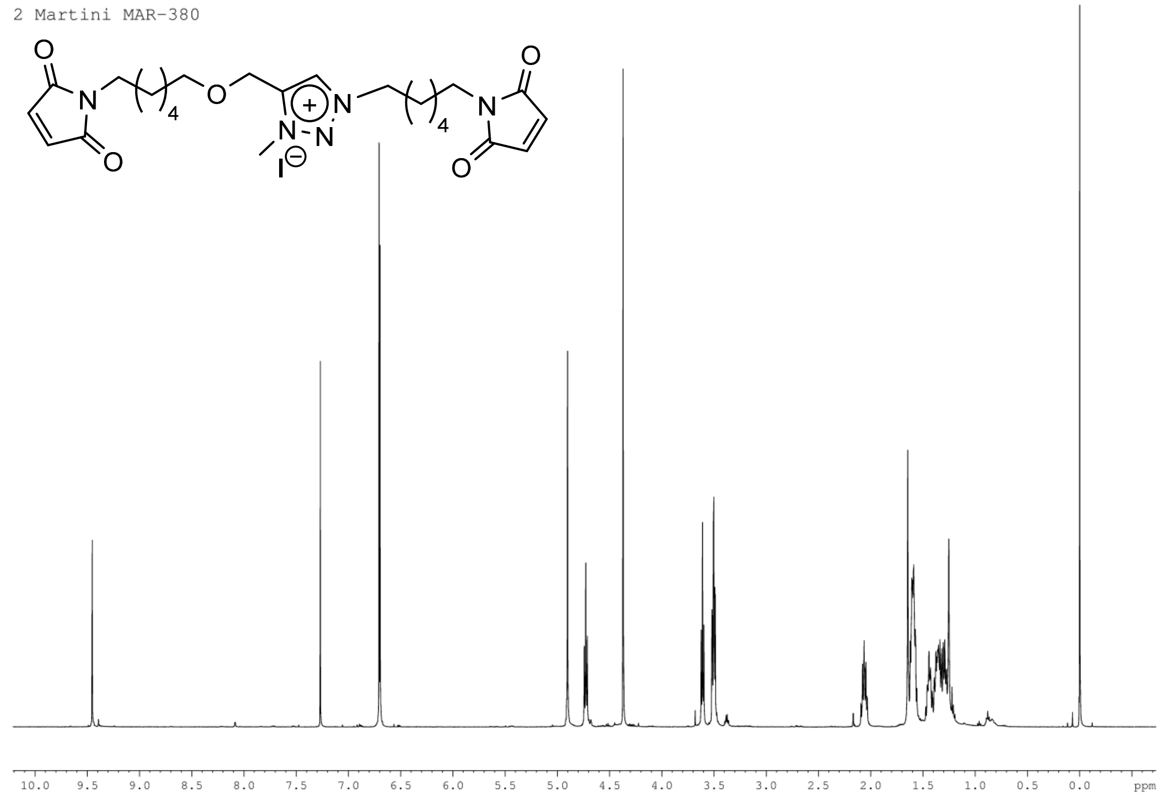

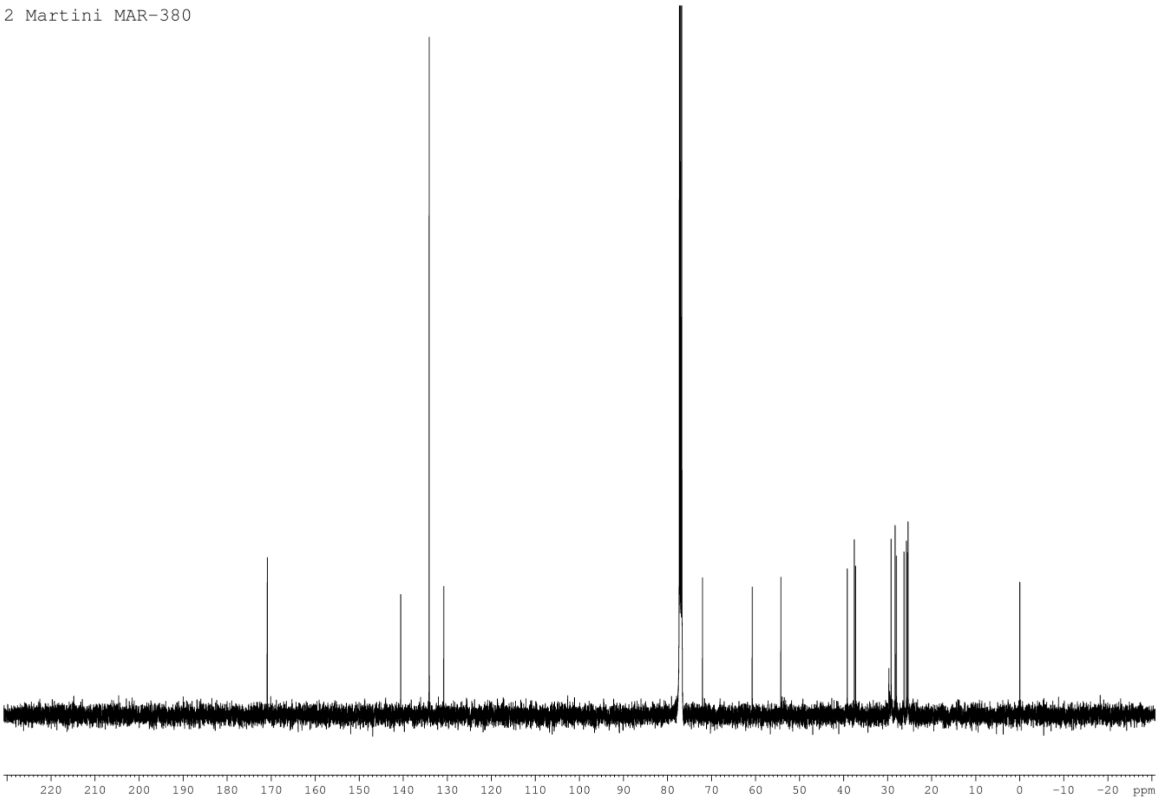

1-[8-(2,5-Dioxo-2,5-dihydro-1H-pyrrol-1-yl)octyl]-4-({[8-(2,5-dioxo-2,5-dihydro-1H-pyrrol-1-yl)octyl]oxy}methyl)-3-methyl-1H-1,2,3-triazol-3-ium iodide (Me-(2c)$^{+}$ I$^{−}$)

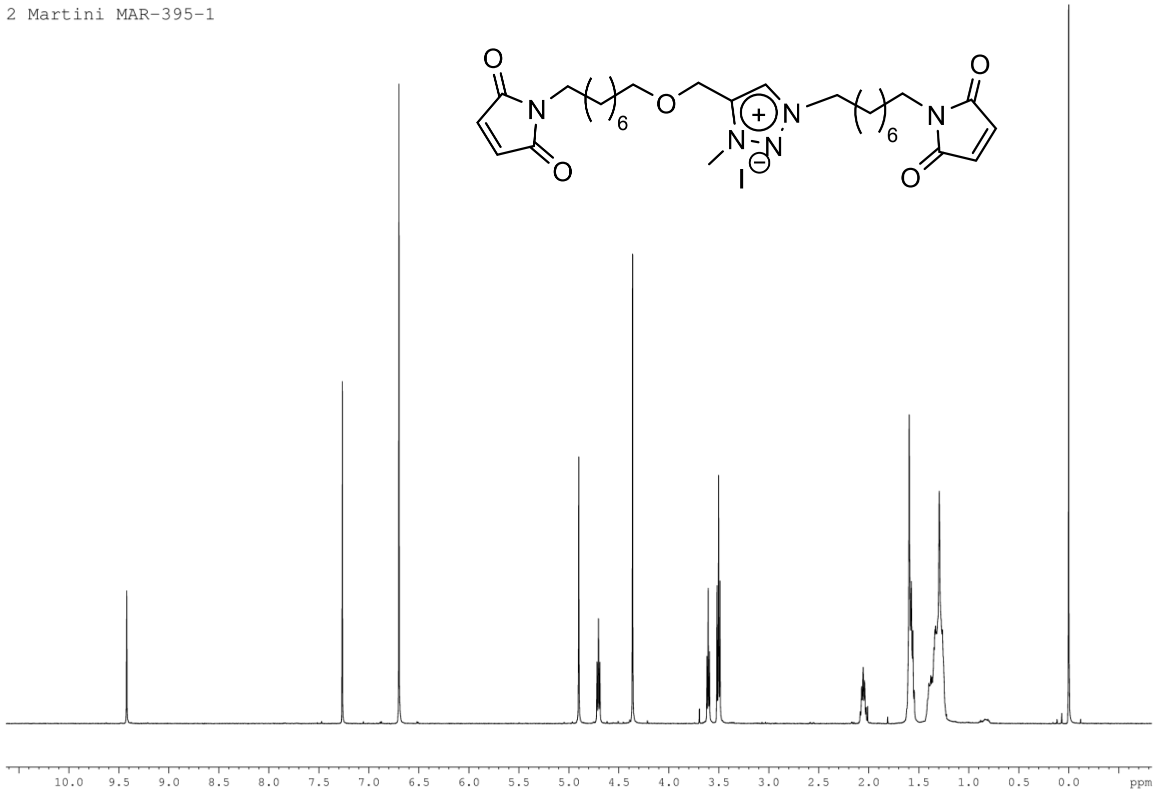

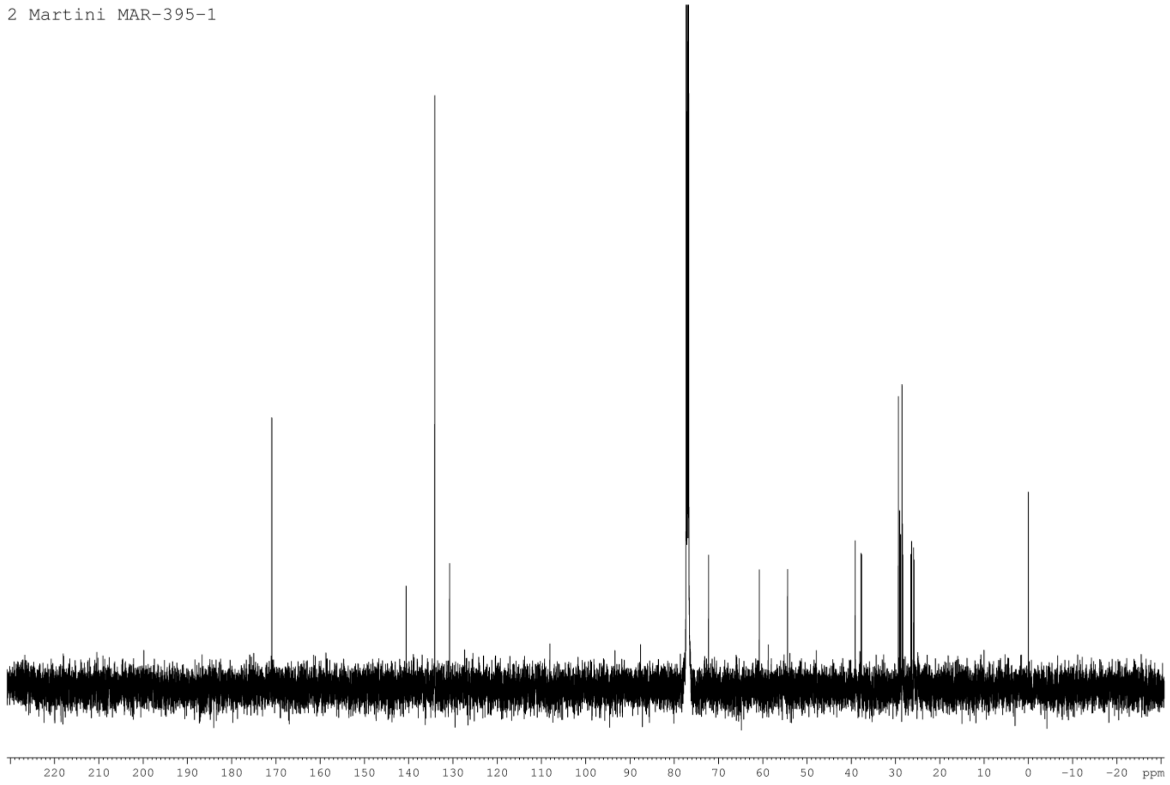

1-[10-(2,5-Dioxo-2,5-dihydro-1H-pyrrol-1-yl)decyl]-4-({[10-(2,5-dioxo-2,5-dihydro-1H-pyrrol-1-yl)decyl]oxy}methyl)-3-methyl-1H-1,2,3-triazol-3-ium iodide (Me-(2d)$^{+}$ I^{-})

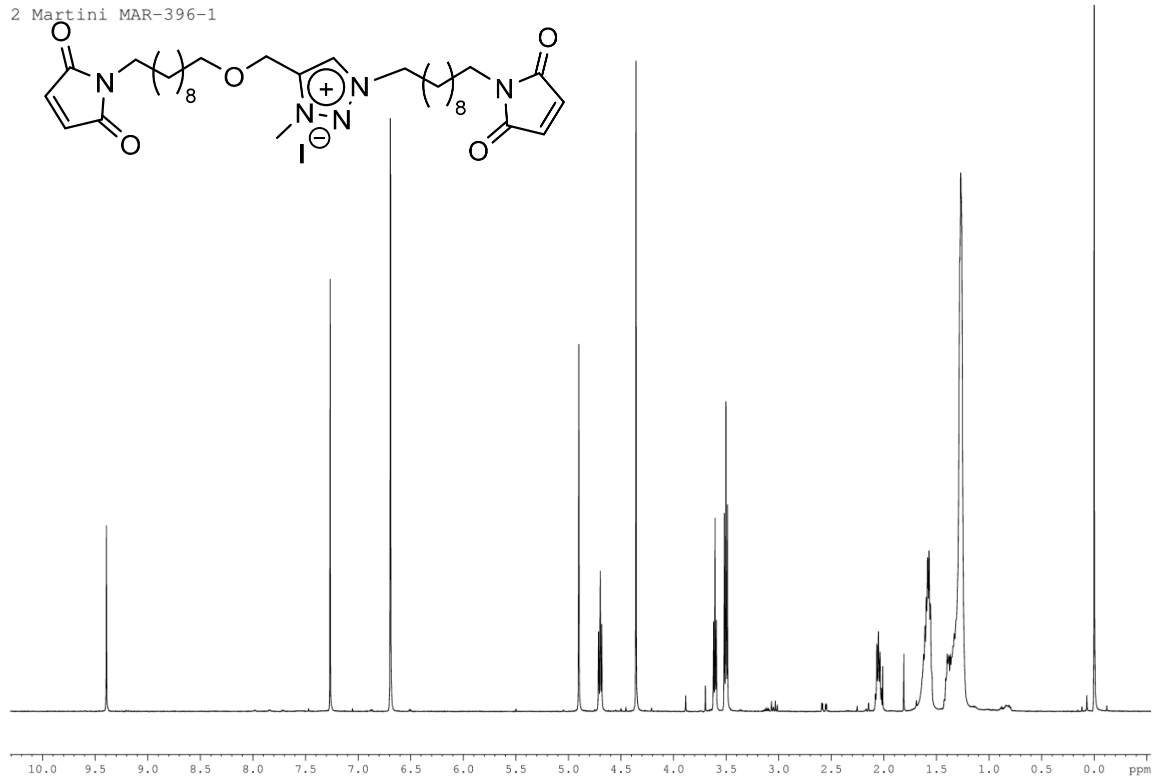

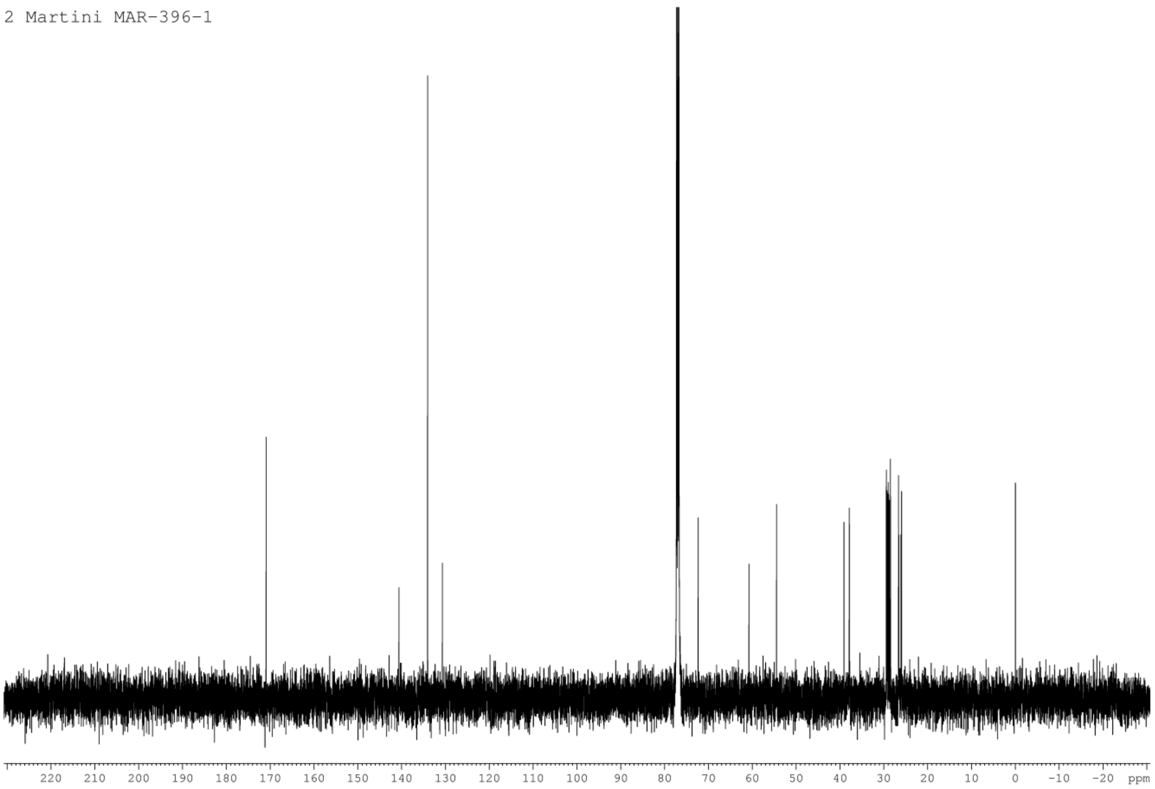

Supplement: Supplementary file 1 [file materials-09-00810-s001.pdf]
